# Spatial Distribution and Ribosome-Binding Dynamics of EF-P in Live *Escherichia coli*

**DOI:** 10.1128/mBio.00300-17

**Published:** 2017-06-06

**Authors:** Sonisilpa Mohapatra, Heejun Choi, Xueliang Ge, Suparna Sanyal, James C. Weisshaar

**Affiliations:** a Department of Chemistry, University of Wisconsin—Madison, Madison, Wisconsin, USA; b Department of Cell and Molecular Biology, Uppsala University, Uppsala, Sweden; Ohio State University; Massachusetts Institute of Technology

**Keywords:** EF-P, binding dynamics, live *E. coli*, superresolution fluorescence

## Abstract

*In vitro* assays find that ribosomes form peptide bonds to proline (Pro) residues more slowly than to other residues. Ribosome profiling shows that stalling at Pro-Pro-X triplets is especially severe but is largely alleviated in *Escherichia coli* by the action of elongation factor EF-P. EF-P and its eukaryotic/archaeal homolog IF5A enhance the peptidyl transfer step of elongation. Here, a superresolution fluorescence localization and tracking study of EF-P–mEos2 in live *E. coli* provides the first *in vivo* information about the spatial distribution and on-off binding kinetics of EF-P. Fast imaging at 2 ms/frame helps to distinguish ribosome-bound (slowly diffusing) EF-P from free (rapidly diffusing) EF-P. Wild-type EF-P exhibits a three-peaked axial spatial distribution similar to that of ribosomes, indicating substantial binding. The mutant EF-P^K34A^ exhibits a homogeneous distribution, indicating little or no binding. Some 30% of EF-P copies are bound to ribosomes at a given time. Two-state modeling and copy number estimates indicate that EF-P binds to 70S ribosomes during 25 to 100% of translation cycles. The timescale of the typical diffusive search by free EF-P for a ribosome-binding site is τ_free_ ≈ 16 ms. The typical residence time of an EF-P on the ribosome is very short, τ_bound_ ≈ 7 ms. Evidently, EF-P binds to ribosomes during many or most elongation cycles, much more often than the frequency of Pro-Pro motifs. Emptying of the E site during part of the cycle is consistent with recent *in vitro* experiments indicating dissociation of the deacylated tRNA upon translocation.

## IMPORTANCE

Ribosomes translate the codon sequence within mRNA into the corresponding sequence of amino acids within the nascent polypeptide chain, which in turn ultimately folds into functional protein. At each codon, bacterial ribosomes are assisted by two well-known elongation factors: EF-Tu, which aids binding of the correct aminoacyl-tRNA to the ribosome, and EF-G, which promotes tRNA translocation after formation of the new peptide bond. A third factor, EF-P, has been shown to alleviate ribosomal pausing at rare Pro-Pro motifs, which are translated very slowly without EF-P. Here, we use superresolution fluorescence imaging to study the spatial distribution and ribosome-binding dynamics of EF-P in live *E. coli* cells. We were surprised to learn that EF-P binds to and unbinds from translating ribosomes during at least 25% of all elongation events; it may bind during every elongation cycle.

## INTRODUCTION

During the elongation phase of protein synthesis in prokaryotes, ribosomes are assisted by at least two cofactors that bind and unbind during each translation cycle (1). EF-Tu mediates the proper binding of the correct aminoacyl-tRNA (aa-tRNA) to the ribosomal A site. EF-G promotes the tRNA translocation step that follows formation of a new peptide bond between aminoacyl-tRNA in the A site and peptidyl-tRNA in the P site. *In vitro* translation assays have found that Pro is incorporated into peptides more slowly than other residues (2–4). This can lead to translational pausing (ribosome stalling), which in turn can cause premature termination of the elongation phase before a complete protein has been synthesized (2–5).

**FIG 1:**
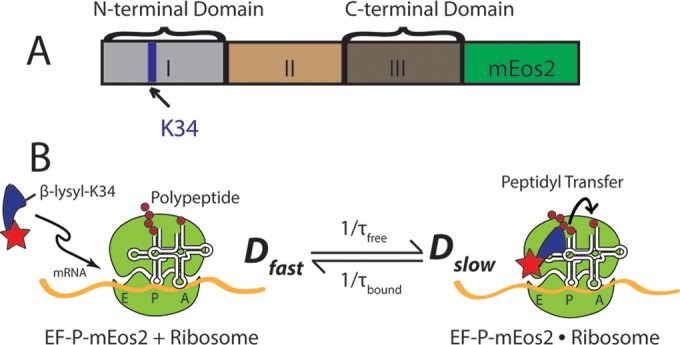
(A) Organization of the different domains of the *E. coli* EF-P protein with C-terminal fusion to mEos2, a fluorophore. The key residue Lys-34 is marked with an arrow. (B) Simple two-state kinetics scheme for EF-P–mEos2 binding to and dissociation from a 70S ribosome with empty E site. Positions of Lys-34 at the N terminus and mEos2 (red star) at the C terminus are depicted schematically. The ribosome-bound species (*D*_slow_) diffuses much more slowly than free EF-P (*D*_fast_). Binding and dissociation rate constants are 1/τ_free_ and 1/τ_bound_, respectively.

Ribosome stalling is known to occur especially frequently at consecutive Pro codons (Pro-Pro motifs), presumably due to geometric constraints imposed by the cyclic structure of proline. *In vitro* kinetics studies show that a third elongation factor, EF-P, helps to alleviate pausing at Pro-Pro motifs (4, 6–8). EF-P is the only known cofactor that accelerates the peptide bond formation step of the translation elongation cycle (9, 10). The size and shape of the 21-kDa EF-P mimic those of tRNA (11). A crystal structure reveals that *Thermus thermophilus* EF-P binds at the interface of the 30S and 50S subunits, between the E and P sites of the ribosome and in close proximity to the peptidyl transfer center (see Fig. 1) (12). Posttranslational β-lysylation at residue Lys-34 and an empty ribosomal E site are evidently prerequisite to EF-P binding and activity (1, 4, 13).

In prokaryotes, EF-P is nonessential, but *efp* mutant strains exhibit pleiotropic phenotypes, including slower growth, loss of membrane integrity, and increased sensitivity to antibiotics (1, 14, 15). Early kinetics and ribosome profiling studies have investigated the nature of stalling sites that are alleviated by EF-P (4, 6–8). Distinct Z-Pro-Pro-X codon sequences have been shown to induce translational stalling in *efp* mutant strains. The stalling strength varied substantially depending on the identity of the codon just downstream (X) or just upstream (Z) of Pro-Pro (6, 7). In addition, EF-P has been shown to alleviate stalling at certain sequences that do not contain a Pro-Pro duet (8, 16).

Here, we use superresolution fluorescence microscopy (17–19) to locate and track single EF-P copies in live *Escherichia coli* cells for the first time. The gene *efp* is replaced in the chromosome with the gene *efp*-*mEos2* for the fluorescent variant. A weak laser at 405 nm photoswitches a sparse set of EF-P–mEos2 molecules from a green fluorescent state to an orange fluorescent state. A probe laser at 561 nm is used to locate and track one or two copies at a time with ~60-nm spatial resolution and 2-ms time resolution. Ribosome-bound copies of EF-P generally exhibit slower diffusion than free copies, as depicted schematically in Fig. 1.

The new results provide the first *in vivo* information about the spatial distribution and ribosome-binding dynamics of EF-P. We provide estimates of the fraction of EF-P that is bound to ribosomes at a given time, the timescale of the diffusive search for empty ribosome E sites, and the duration of typical transient binding events. Our numerical estimates from two-state modeling indicate that EF-P binds to 70S ribosomes during at least one of every four elongation cycles but typically for a very short time period (τ_bound_ ≈ 7 ms). Such EF-P binding events evidently occur much more frequently than previously suspected. This suggests that the E site becomes empty and thus open to EF-P binding during part of each elongation cycle. That inference in turn is consistent with *in vitro* experiments indicating that the deacylated tRNA is released from the E site rapidly upon translocation (20–22). The new data are inconsistent with a concerted mechanism of tRNA progression through the A, P, and E sites, i.e., a mechanism in which the E site is never completely empty. However, our data do not resolve the question of whether or not accommodation of a new aa-tRNA at the A site is prerequisite to release of the deacylated tRNA (20–25).

## RESULTS

### Comparison of axial spatial distributions of EF-P and ribosomes in normal growth.

We have fused the gene coding for the photoswitchable fluorescent protein mEos2 to the C terminus of the endogenous *efp* gene in *E. coli* MG1655 and then moved the fusion to the VH1000 background for further study (Fig. 1; see also Table S1 in the supplemental material) (26). Under our growth conditions, the doubling time of the modified strain expressing EF-P–mEos2 is 1.3 times longer than that of the VH1000 background strain, suggesting that the labeling does not greatly affect the functionality of *efp* or the ribosome. For comparison, deletion of *efp* increases the doubling time by a factor of two, in both *E. coli* and *Salmonella* (15). EF-P consists of three domains (Fig. 1). It binds to the ribosome between the P and the (otherwise empty) E site (4, 12). The Lys-34 residue in domain I, which is responsible for the functionality of EF-P, lies near the N terminus of the protein and contacts the P-site tRNA. The C-terminal domain of EF-P interacts with the P-site tRNA and with the 30S ribosomal subunit (12). Based on the crystal structure (12), labeling of EF-P with mEos2 at the C terminus may cause a steric clash between mEos2 and the 30S subunit. However, this evidently does not greatly perturb ribosome functionality, as judged by the moderate 30% increase in doubling time for the mutant strain compared with the wild-type (WT) VH1000 strain. 

10.1128/mBio.00300-17.9 TABLE S1  (A) Oligonucleotides used in construction of strains described in the text. (B)** **Strains used for imaging and doubling times at 30°C in EZ rich defined medium (EZRDM). Download TABLE S1, DOCX file, 0.01 MB. Copyright © 2017 Mohapatra et al. 2017 Mohapatra et al. This content is distributed under the terms of the Creative Commons Attribution 4.0 International license.

Our goal is to use diffusion data and spatial distributions to distinguish EF-P copies bound to 70S (translating) ribosomes from free EF-P copies. Free, monomeric EF-P–mEos2 has a mass of 47 kDa (11, 26). An unbound species of that mass would have a diffusion coefficient of roughly 4 to 10 µm^2^/s in the *E. coli* cytoplasm (27). In contrast, EF-P bound to 70S ribosomes would diffuse much more slowly, with a diffusion coefficient of ~0.2 μm^2^ s^−1^ (28, 29). Bound EF-P should also exhibit a three-peaked, ribosome-like axial distribution of locations within the cytoplasm (30). We imaged EF-P–mEos2 molecules in live cells by photoactivating and locating fluorophores (17), connecting locations over multiple frames to form trajectories of individual molecules (31). Details are provided in Materials and Methods and in Text S1 in the supplemental material. To enable efficient superresolution imaging of rapidly diffusing molecules, the exposure time was 2 ms/frame with continuous laser illumination. The number of switched-on copies per cell was limited to 0 to 2 molecules per frame to avoid spatial overlap of the images.

10.1128/mBio.00300-17.1 TEXT S1 The supplemental text file describes the data analysis and fitting procedures in detail. Download TEXT S1, DOCX file, 2.5 MB. Copyright © 2017 Mohapatra et al. 2017 Mohapatra et al. This content is distributed under the terms of the Creative Commons Attribution 4.0 International license.

**FIG 2:**
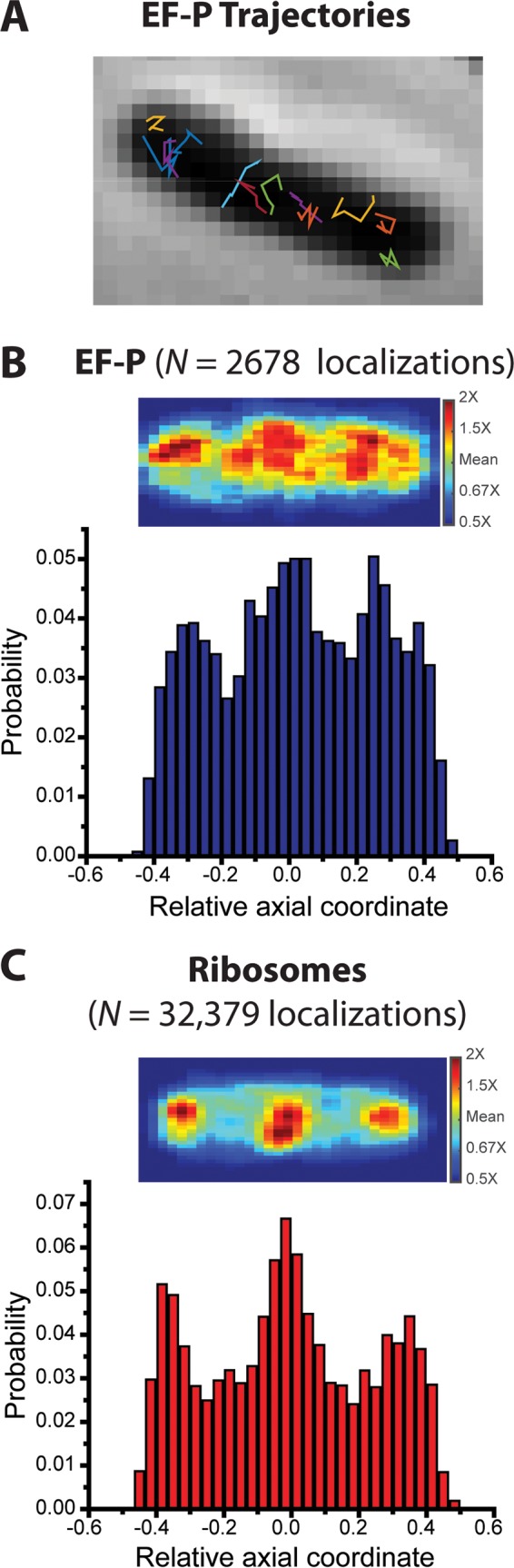
(A) Phase-contrast image of an example cell overlaid with trajectories of single EF-P–mEos2 copies. Imaging at 2 ms/frame. (B) (Top) Localization probability density heat map of 2,678 EF-P–mEos2 copies imaged at 2 ms/frame in different cells of 3.5 to 4.5 µm in length. Each location is placed on a common scale of relative axial position. Only molecules that lasted at least 7 frames contribute to the distribution. (Bottom) Distribution of axial projections on the same relative scale. (C) (Top) Localization probability density map of 32,000 ribosome copies (30S labeled by mEos2). Same imaging conditions as t hose for EF-P–mEos2. (Bottom) Distribution of axial projections on the same relative scale.

We studied 120 cells whose tip-to-tip cell length varied from 3.2 to 8.4 µm. We sorted the cells by length into bins of 1-µm width. Some typical trajectories are shown in Fig. 2A. To obtain the axial distribution of EF-P (Fig. 2B), we combined axial locations of molecules from the 60 cells of 3.5 to 4.5 µm in length. For comparison with the detailed trajectory analysis presented below, all locations measured in trajectories that last at least seven frames or longer contribute to the data shown in Fig. 2B. The results are very similar when all locations from all trajectories are included. To account for cell length variability within the chosen bin width, the axial locations are scaled to the range −0.5 to +0.5, with 0 representing the cell center and ±0.5 representing the locations of the two cell tips.

For direct comparison, we also performed single-molecule imaging of 30S subunits of ribosomes labeled by an S2-mEos2 construct expressed from the chromosome. Details regarding the construction of this strain were described previously (32). As inferred from the unchanged growth rate (33), labeling with mEos2 does not affect the functionality of S2, a small ribosomal protein that binds to the exterior of the 30S subunit. The spatial distribution of ribosomes (30S-mEos2 labeling) was obtained by imaging a combination of 30S subunits and translating 70S ribosomes at the same 2-ms intervals. The same scaling procedure was applied to localizations of ribosome trajectories that lasted at least seven frames in cells of 3.5 to 4.5 µm in length (Fig. 2C).

The ribosome axial distribution exhibits three distinct peaks, consistent with our previous studies (30). As before, translating ribosomes are strongly segregated from the nucleoid regions. Comparing Fig. 2B and C, we see that the axial distribution of EF-P also exhibits three peaks, qualitatively similar to the ribosomes. This is consistent with some degree of binding of EF-P to ribosomes. However, the peak-to-trough ratio in the axial distribution of EF-P is not as large. Evidently, only a fraction of the EF-P copies reside in the ribosome-rich regions at any given time. Detailed diffusion measurements described below quantify the fraction of EF-P associated with ribosomes.

### Drug effects on axial distributions: Cam and rifampin (Rif).

*In vitro* measurements have suggested that translational pausing and the resulting empty ribosome E site are prerequisites for binding of EF-P between the E and P sites of ribosomes (1). To further understand the binding of EF-P to ribosomes in live cells, we investigated the axial distribution of EF-P–mEos2 after treatment with the translation-halting drug chloramphenicol (Cam). Cam binds between the P and A sites of the 70S ribosome, inhibiting peptidyl transfer and stalling translating ribosomes in place (34–36). Binding of Cam presumably leaves the space between E and P sites more often available for binding by EF-P, due to dissociation of tRNA from the E site after translation is halted.

Our previous studies have shown that after treatment with Cam, very strong nucleoid-ribosome segregation occurs (32). We generated the axial distributions of EF-P–mEos2 and of 30S-mEos2 at *t* = 30 min after treatment with 200 μg/ml of Cam (Fig. S1A and B). The average cell length decreases dramatically upon Cam treatment (32). To obtain sufficient data, cell lengths of 2.5 to 3.5 µm were selected. After Cam treatment, the ribosomes show a very clear two-peaked axial distribution, consistent with previous work (30). The EF-P axial distribution closely resembles that of ribosomes (Fig. S1B), indicating that EF-P strongly associates with 70S ribosomes whose translation has been halted by Cam.

10.1128/mBio.00300-17.2 FIG S1  (A) (Top) Localization probability density heat map of 2,220 EF-P–mEos2 copies imaged at 2 ms/frame in different cells of 2.5 to 3.5 µm in length after 30 min of chloramphenicol treatment. Each location is placed on a common scale of relative axial position. Only molecules that lasted at least 7 frames contribute to the distribution. (Bottom) Distribution of axial projections on the same relative scale. (B) Same as panel A, but for ribosomes imaged after Cam treatment. (C) Same as panel A, but for EF-P–mEos2 molecules imaged in cells of 3.5 to 4.5 μm in length after 3 h of rifampin treatment. (D) Same as panel C, but for ribosomes imaged after Rif treatment. Download FIG S1, TIF file, 44.6 MB. Copyright © 2017 Mohapatra et al. 2017 Mohapatra et al. This content is distributed under the terms of the Creative Commons Attribution 4.0 International license.

Rifampin (Rif) halts transcription and leads to depletion of mRNA-bound 70S ribosomes. After 3 h of treatment with Rif, most ribosomal subunits are free (32). These free 30S and 50S subunits mix with the expanded nucleoids, and the 30S spatial distribution becomes essentially homogeneous throughout the cytoplasm (30, 37). We used the same imaging conditions for EF-P–mEos2 and 30S-mEos2 molecules at *t* = 3 h after treatment with 300 μg/ml of Rif. Like that of 30S, the spatial distribution of EF-P molecules shows little or no axial segregation (Fig. S1C and D). However, the two rather homogeneous axial distributions do not determine whether EF-P is unbound or associates with 50S or 30S ribosomal subunits. Diffusion measurements after drug treatment shed light on this question.

### Axial distribution of EF-P^K34A^ expressed from a plasmid in normal growth.

*In vitro* studies have shown that Lys-34 of EF-P is essential for functionality (4, 13, 15). Deletion of EF-P or expression of mutated, nonfunctional EF-P increases the doubling time by a factor of 2 and induces other growth defects as well (1, 14, 15). To study the importance of Lys-34 in live *E. coli*, we constructed a strain containing an inducible plasmid that expresses the mutant form EF-P^K34A^–mEos2 (Table S1A). Fully functional, unlabeled EF-P continues to be expressed from the chromosome to ensure normal growth rate and cell functionality. Accordingly, the doubling time for the mutant strain is almost identical to that of the VH1000 parent strain (Table S1B).

**FIG 3:**
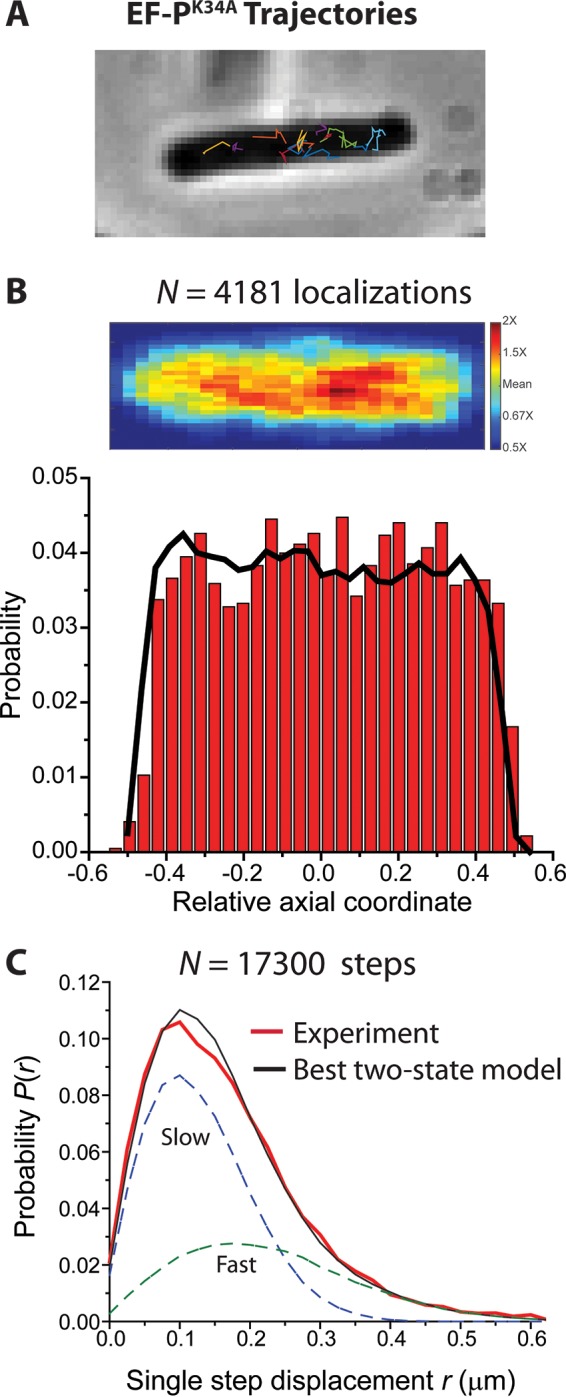
(A) Phase-contrast image of an example cell overlaid with trajectories of single mutant EF-P^K34A^–mEos2 copies. Imaging at 2 ms/frame. (B) (Top) Localization probability density heat map of 4180 EF-P^K34A^–mEos2 copies imaged at 2 ms/frame in different cells of 3.5 to 4.5 µm in length. Each location is placed on a common scale of relative axial position. Only molecules that lasted at least 7 frames contribute to the distribution. (Bottom) Distribution of axial projections on the same relative scale. For comparison, axial distribution of molecules uniformly filling a spherocylinder of 0.9-µm diameter and 4-µm length is shown in black. (C) Red, experimental probability distribution of 17,300 single-step displacements taken by EF-P^K34A^–mEos2 molecules in 2 ms. Black, the best unconstrained fit to a static two-state model (without transitions). Model parameters: *D*_slow_ = 3.2 µm^2^/s (σ_slow_ = 50 nm), *f*_slow_ = 0.65, *D*_fast_ = 9.7 µm^2^/s (σ_fast_ = 90 nm), *f*_fast_ = 0.35, with χ_ν_^2^ = 2.0. The slow and fast components are shown as dashed lines as labeled.

We studied the axial distribution of EF-P^K34A^–mEos2 after induction of the gene under normal growth conditions. The methodology was the same as that for the EF-P–mEos2 imaging experiments. The EF-P^K34A^–mEos2 trajectories (Fig. 3A) are generally more extended than those of EF-P–mEos2. The axial distribution of EF-P^K34A^–mEos2 (Fig. 3B) is not ribosome-like, in sharp contrast to the three-peaked distribution of normal EF-P–mEos2 (Fig. 2B). Instead, the EF-P^K34A^–mEos2 molecules are distributed essentially uniformly throughout the cell. For comparison, we simulated the axial distribution for a uniformly filled spherocylinder of 4-µm length and 0.9-µm diameter with the same number of molecules as the experimental axial distribution (solid black line in Fig. 3B). The experimental axial distribution of EF-P^K34A^–mEos2 closely resembles the simulated homogeneous distribution; the wild-type EF-P–mEos2 distribution (Fig. 2B) does not. This indicates that EF-P^K34A^ associates with translating ribosomes significantly less than with wild-type EF-P, in agreement with previous binding studies *in vitro* (4).

To ensure that the homogeneous distribution of EF-P^K34A^–mEos2 is not an artifact of overexpression from a plasmid, we constructed a control strain (SM4, Table S1A) in which the wild-type EF-P–mEos2 is expressed from an analogous plasmid alongside the expression of unlabeled, endogenous EF-P from the chromosome. The gene induction conditions are the same in the two experiments. The axial distribution of wild-type EF-P–mEos2 expressed upon induction from the plasmid is again three-peaked, like that of ribosomes (Fig. S2). EF-P–mEos2 expressed from the plasmid also exhibits diffusive behavior very similar to that of EF-P expressed endogenously from the chromosome. This indicates that the observed behavior of EF-P^K34A^–mEos2 is not an artifact of expression from a plasmid.

10.1128/mBio.00300-17.3 FIG S2  Localization probability density maps of EF-P–mEos2 expressed from plasmid under normal growth conditions. A composite of images taken at 2 ms/frame from cells with lengths between 3.5 and 4.5 µm. Only molecules that lasted at least 7 frames contribute to the axial distribution. The axial distribution shows a three-peak distribution similar to that of ribosomes under the same imaging conditions. Download FIG S2, TIF file, 14.6 MB. Copyright © 2017 Mohapatra et al. 2017 Mohapatra et al. This content is distributed under the terms of the Creative Commons Attribution 4.0 International license.

### Diffusion of EF-P expressed from the chromosome and of EF-P^K34A^ expressed from a plasmid.

The axial distribution of EF-P–mEos2 gives only qualitative information about the fraction of EF-P that is associated with translating ribosomes *in vivo* and no information about the dynamics of the association process. To investigate the diffusive behavior of EF-P in live cells under normal growth conditions, we chose trajectories that lasted at least seven frames, similar to those used for generating the axial distributions. The mean diffusion coefficient <*D*> can be estimated from a plot of the two-dimensional mean square displacement versus lag time, MSD(τ), using the slope of the first two data points (Fig. S4A). The MSD(τ) plot is a weighted average over different diffusive states of EF-P molecules. The result <*D*> = 3.4 µm^2^/s for EF-P–mEos2 is somewhat smaller than our rough estimate for a freely diffusing molecule of similar size (4 to 10 µm^2^/s) but much larger than <*D*> for ribosomes under similar fast-imaging conditions (0.5 µm^2^/s, Fig. S4A). Combined with the axial distribution results, this suggests that only a fraction of EF-P copies are associated with the translating ribosomes at a given time. 

**FIG 4:**
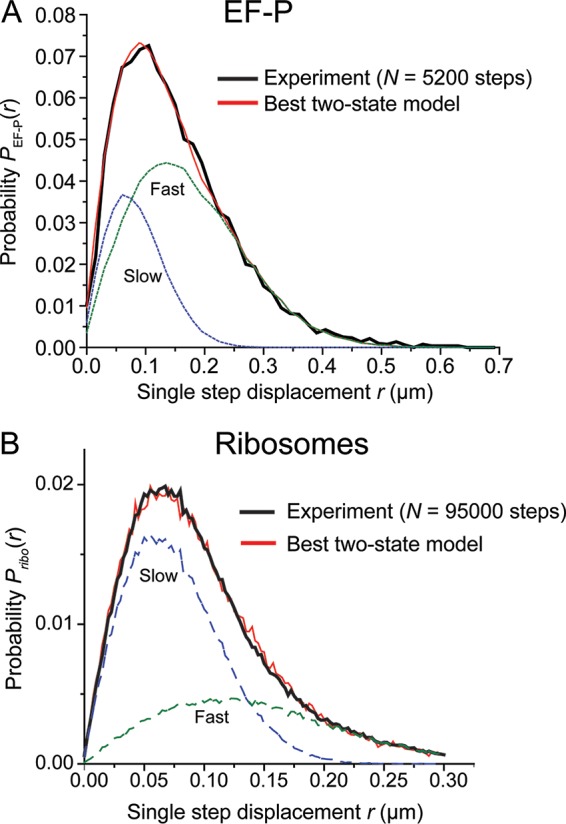
(A) Black, experimental probability distribution of single-step displacements taken by EF-P–mEos2 molecules in 2 ms. Red, the best fit to a static two-state model (without transitions) with *D*_slow_ constrained. Model parameters: *D*_slow_ = 0.2 µm^2^/s (σ_slow_ = 50 nm), *f*_slow_ = 0.30, *D*_fast_ = 4.3 µm^2^/s (σ_fast_ = 75 nm), *f*_fast_ = 0.7, with a χ_ν_^2^ = 1.0. The individual slow and fast components are shown in dashed lines as labeled. (B) Black, experimental probability distribution of single-step displacements taken by ribosomes (30S labeled with mEos2) in 2 ms. Red, the best unconstrained fit to a static two-state model (without transitions). Model parameters: *D*_slow_ = 0.20 µm^2^/s (σ_slow_ = 40 nm), *f*_slow_ = 0.65, *D*_fast_ = 0.8 µm^2^/s (σ_fast_ = 75 nm), *f*_fast_ = 0.35, with χ_ν_^2^ = 2.3. The slow and fast components are shown as dashed lines as labeled.

To quantify the fraction of ribosome-bound EF-P, the chosen trajectories were divided into individual steps with Δ*t* = 2 ms between camera frames, in hopes of isolating short time intervals during which EF-P remains in one diffusive state. The resulting distribution of experimental single-step displacements, *P*_EF-P_(*r*), is shown for 5,200 individual steps in Fig. 4A. The analogous plot for 30S-labeled ribosomes imaged under the same conditions, *P*_ribo_(*r*), is shown in Fig. 4B.

We analyze *P*(*r*) distributions by comparison to the results of a large number of simulated random walk trajectories that incorporate dynamic localization error σ and confinement within a spherocylinder that mimics the dimensions of an *E. coli* cell. Details are provided in Text S1. For each chosen diffusion coefficient *D* and measurement error σ, the simulations provide a numerical function we call *P*_model_(*r*;*D*). We attempt to fit the experimental distribution *P*(*r*) in a least-squares sense to a single population or to a weighted average of two static populations. The goodness of fits was judged by the reduced chi-square statistic χ_ν_^2^ (38). For a one-state model, the only fitting parameter is *D*. For unconstrained models, including two static (nonexchanging) states, the fitting function is the linear combination *P*_model_(*r*) = *f*_slow_*P*(*r*;*D*_slow_) + (1 – *f*_slow_)*P*(*r*;*D*_fast_). The three fitting parameters are *D*_fast_, *D*_slow_, and the fractional population *f*_slow_, which in turn fixes *f*_fast_ = (1 – *f*_slow_).

First, we analyzed the single-step ribosome distribution *P*_ribo_(*r*) from 15,800 6-step trajectories (Fig. 4B; Table 1). All single-population fits were poor. The best fit to a sum of two static populations yielded *f*_slow_ = 0.65, *D*_slow_ = 0.2 µm^2^/s (presumably the 70S ribosomes engaged in translation as polysomes of variable size), *f*_fast_ = 0.35, and *D*_fast_ = 0.8 µm^2^/s (presumably the free 30S subunits). The minimum goodness-of-fit parameter (reduced chi-square) was χ_ν_^2^ = 2.3, indicating a reasonable fit given the noise in both experimental and model histograms (see Text S1 for details). The MSD plot yielded a mean diffusion coefficient for ribosomes under imaging at 2 ms/frame of *D*_ribo_ = 0.5 µm^2^/s (Fig. S4A), consistent with previous measurements by English et al. under similarly fast imaging conditions (28). The value *D*_slow_ = 0.2 µm^2^/s is similar to that used by Plochowietz et al. to identify tRNA associated with translating ribosomes (29).

**TABLE 1:** Summary of two-state, best-fit results for *P*(*r*) for different species^a^

Species (expression mode)	*D*_slow_ (μm^2^/s)	*D*_fast_ (μm^2^/s)	*f*_slow_
Ribosome (chromosome; 30S)	0.2	0.8	0.65
EF-P (chromosome)	Constrained to 0.2	4.3 ± 1.0	0.30 ± 0.10
EF-P after Cam (chromosome)	Constrained to 0.2	1.2	0.45
EF-P after Rif (chromosome)	4.6	8	0.55
EF-P^K34A^^b^ (plasmid)	3.2	9.7	0.65

a See Text S1 in the supplemental material for details of fitting procedure.

b Unlabeled WT EF-P is natively expressed from the chromosome.

For the experimental *P*_EF-P_(*r*) distribution of Fig. 4A, we again found no good one-state fits. Then, we tested a two-state model of EF-P with no exchange between states on the 2-ms timescale of the single steps. The two states were a slowly diffusing, ribosome-bound state (*D*_slow_) and a rapidly diffusing, free state (*D*_fast_), with population fractions *f*_slow_ and *f*_fast_. The large measurement error precludes accurate determination of *D*_slow_, as detailed in Text S1. The root mean square displacement of a ribosome-bound (slow) EF-P copy is comparable to the typical static localization error of our experiments (~60 nm). We therefore constrained *D*_slow_ = 0.2 μm^2^ s^−1 ^(to match that of ribosomes) and optimized *f*_slow_ and *D*_fast_ to obtain the best fit to the experimental *P*_EF-P_(*r*). Our best parameter estimates are *f*_slow_ = 0.30 ± 0.10, *D*_fast_ = 4.3 ± 1.0 μm^2^ s^−1^, and *f*_fast_ = 0.70 ± 0.10, with the best χ_ν_^2^ = 1.0, indicating a good fit (Table 1). This fit is compared with the data in Fig. 4A. Error estimation for the parameters is described in Text S1. We assign the slowly diffusing EF-P fraction as the copies bound to translating 70S ribosomes, consistent with the axial spatial distribution. The rapidly diffusing fraction is attributed to free EF-P.

Under Cam treatment, we expect more E sites available for EF-P binding than in normal growth. The best-fit two-state *P*_model_(*r*) was obtained for *f*_slow_ = 0.45 (with *D*_slow_ constrained to 0.2 µm^2^/s) and *f*_fast_ = 0.55, *D*_fast_ = 1.2 µm^2^/s, yielding χ_ν_^2^ = 1.2 (Table 1; Fig. S3A). Cam treatment apparently increases the fraction of slowly diffusing EF-P and also decreases *D*_fast_ from 4.3 to 1.2 µm^2^/s. The more rapidly diffusing component under Cam treatment is likely experiencing transient binding events to 70S ribosomes, consistent with the suggestion that Cam induces more binding opportunities.

10.1128/mBio.00300-17.4 FIG S3  (A) Red, experimental probability distribution of single-step displacements taken by EF-P–mEos2 molecules in 2 ms after 30 min of chloramphenicol treatment. Black, the best fit to a static two-state model (without transitions) with *D*_slow_ constrained. Model parameters: *D*_slow_ = 0.2 µm^2^/s (σ_slow_ = 50 nm), *f*_slow_ = 0.45, *D*_fast_ = 1.2 µm^2^/s (σ_fast_ = 90 nm), *f*_fast_ = 0.55, with χ_ν_^2^ = 1.2. The slow and fast components are shown as dashed lines as labeled. (B) Red, experimental probability distribution of single-step displacements taken by EF-P–mEos2 molecules in 2 ms after 3 h of rifampin treatment. Black, the best unconstrained two-state model fit. Model parameters: *D*_slow_ = 4.6 µm^2^/s (σ_slow_ = 75 nm), *f*_slow_ = 0.55, *D*_fast_ = 8 µm^2^/s (σ_fast_ = 150 nm), *f*_fast_ = 0.45, with χ_ν_^2^ = 1.5. The individual slow and fast components are shown in dashed lines of blue and green, respectively. Download FIG S3, TIF file, 81.8 MB. Copyright © 2017 Mohapatra et al. 2017 Mohapatra et al. This content is distributed under the terms of the Creative Commons Attribution 4.0 International license.

10.1128/mBio.00300-17.5 FIG S4  (A) Mean square displacement plot, MSD(τ), for EF-P–mEos2 (red circles) and for ribosomes (30S-mEos2 labeling, black circles). Trajectories are truncated to six steps; error estimates are ±1 σ of the MSD values. Lines are drawn through the first two data, yielding diffusion coefficient estimates of 3.5 µm^2^/s and 0.5 µm^2^/s, respectively. (B) Mean square displacement plot, MSD(τ), for EF-P–mEos2 after 3 h of treatment with Rif (red circles) and the mutant form EF-P^K34A^–mEos2 under normal growth conditions (black circles). Trajectories are truncated to six steps; error estimates are ±1 σ of the MSD values. Lines are drawn through first two data, yielding diffusion coefficient estimates of 6.7 µm^2^/s and 4.6 µm^2^/s, respectively. Download FIG S4, TIF file, 25.4 MB. Copyright © 2017 Mohapatra et al. 2017 Mohapatra et al. This content is distributed under the terms of the Creative Commons Attribution 4.0 International license.

In contrast, under rifampin treatment, we obtained the best-fit *P*_model_(*r*) for two static populations with *f*_slow_ = 0.55, *D*_slow_ = 4.6 µm^2^/s, *f*_fast_ = 0.45, and *D*_fast_ = 8 µm^2^/s, with χ_ν_^2^ = 1.5 (Table 1; Fig. S3B). Fits of the experimental *P*(*r*) to a single population yielded much larger values of χ_ν_^2^. Both diffusion coefficients are much faster than those of translating 70S ribosomes and of free 30S and 50S subunits. The diffusion data show no clear evidence of EF-P association with nontranslating 30S or 50S ribosomal subunits. The corresponding homogenous axial distribution of EF-P after Rif treatment (Fig. S1C) is evidently due to essentially freely diffusing EF-P.

For EF-P^K34A^–mEos2, a similar procedure resolved the experimental *P*(*r*) into two static populations with *f*_slow_ = 0.65, *D*_slow_ = 3.2 µm^2^/s, *f*_fast_ = 0.35, and *D*_fast_ = 9.7 µm^2^/s, with χ_ν_^2^ being 1.96 (Fig. 3C; Table 1). This is similar to the result for wild-type EF-P after Rif treatment, in that there is no clear evidence of a slowly diffusing component of EF-P^K34A^ that moves like translating ribosomes. Based on the spatial distribution as well as the diffusion measurements, we see almost no evidence of EF-P^K34A^ associating with the translating ribosomes. This is consistent with *in vitro* studies (4) that have shown a 30-fold reduction in the binding affinity of EF-P^K34A^ with ribosomes compared with wild-type EF-P. With *D*_slow_ constrained to the ribosome-like value of 0.2 μm^2^ s^−1^, the best fit is qualitatively poor and the χ_ν_^2^ value doubles. Evidently, EF-P^K34A^ interacts much more weakly with translating ribosomes than does wild-type EF-P, consistent with the observed homogeneous spatial distribution. The value *D*_slow_ = 3.2 µm^2^/s admits the possibility of weaker, shorter-lived interactions with 70S ribosomes.

### Dynamics of EF-P association with ribosomes.

**FIG 5:**
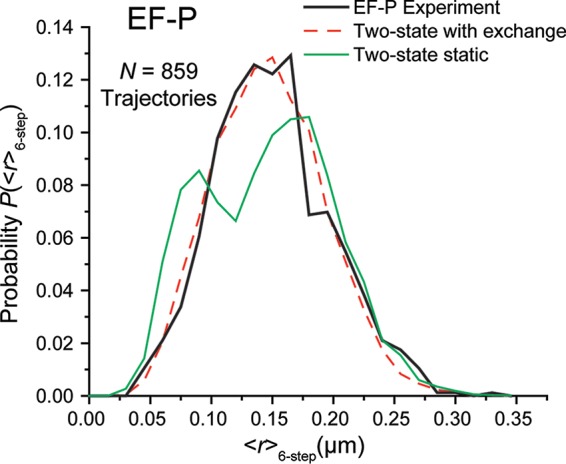
Black, experimental probability distribution of the mean of six consecutive steps from each trajectory for EF-P–mEos2 molecules. Longer trajectories truncated to 6 steps. Red dashed line, best-fit two-state model with binding-unbinding kinetics. Model parameters: *D*_slow_ = 0.2 µm^2^/s (σ_slow_ = 50 nm), *f*_slow_ = 0.3, *D*_fast_ = 4.3 µm^2^/s (σ_fast_ = 75 nm), *f*_fast_ = 0.7, τ_free_ = 16 ms, and τ_bound_ = 7 ms, with χ_ν_^2^ = 1.4. Green line, for comparison, a simulated static two-state model (no transitions) using the same diffusion coefficients and fractions gave χ_ν_^2^ = 10.1.

The fit to the single-step *P*_EF-P_(*r*) distribution assumed two static populations that persist on the 2-ms timescale of single camera frames. Next, we asked whether six-step trajectories (12-ms total duration) could provide information about the timescale of possible transitions between the slow, ribosome-associated state and the fast, freely diffusing state of EF-P. Accordingly, for 859 six-step trajectories of wild-type EF-P–mEos2, we generated the distribution *P*_EF-P_(<*r*>_6-step_), with <*r*>_6-step_ the mean displacement of the six steps. See Text S1 for details. The experimental distribution is shown in Fig. 5. Comparing Fig. 5 and 4, we see that *P*_EF-P_(<*r*>_6-step_) is narrower than *P*_EF-P_(*r*) due to the averaging of six successive displacements.

We simulated *P*_model_(<*r*>_6-step_) distributions for static populations by averaging the displacements from six-step simulated trajectories that included confinement and measurement error. If the EF-P molecules undergo transitions only rarely during a 12-ms trajectory, then the experimental *P*(<*r*>_6-step_) should match the *P*_model_(<*r*>_6-step_) using the same input parameters that gave the best-fit *P*_model_(*r*) distribution. It does not. We simulated 15,000 six-step trajectories, 30% with *D*_slow_ = 0.2 µm^2^/s (4,500 trajectories) and 70% with *D*_fast_ = 4.3 µm^2^/s (10,500 trajectories) in accord with the parameters of the best-fit *P*_model_(*r*) distribution of Fig. 4A. *P*_model_(<*r*>_6-step_) for these static populations exhibits two partially resolved peaks (Fig. 5), unlike the experimental distribution. This suggests that the wild-type EF-P indeed undergoes transitions during the six-step, 12-ms-long trajectories.

In order to estimate the lifetime of the two diffusive states, we introduced two-state, binding/unbinding kinetics into our simulations, according to the work of Das et al. (33, 39). Details are provided in Text S1. We generated large sets of six-step simulated trajectories with *D*_slow_ = 0.2 µm^2^/s, *D*_fast_ = 4.3 µm^2^/s, and τ_free_/τ_bound_ = 7/3; this lifetime ratio is fixed by the fractional populations from the best-fit *P*_model_(*r*) distribution. Here, τ_free_ and τ_bound_ are the lifetimes of the free and ribosome-bound states of EF-P, respectively. While keeping the ratio τ_free_/τ_bound_ constant, we varied τ_free_ from 0.1 ms to 100 ms, calculating 15,000 trajectories in each case. The best fit to the experimental *P*_EF-P_(<*r*>_6-step_) was obtained for τ_free_ = 16 ms and τ_bound_ = 7 ms, with χ_ν_^2^ = 1.4. This fit is compared with the experiment in Fig. 5. Additional calculated distributions *P*_model_(<*r*>_6-step_) for different assumed lifetimes are compared with the experiment in Fig. S6. Based on the quality of the different fits (detailed in Text S1), we take τ_free_ = 16 ± 5 ms and τ_bound_ = 7 ± 2 ms as best estimates of the average lifetime of free EF-P and of EF-P bound to ribosomes, respectively. The typical timescale for an EF-P/ribosome-binding/unbinding cycle is then (τ_bound_ + τ_free_) ≈ 23 ms.

10.1128/mBio.00300-17.6 FIG S5  (A) For EF-P–mEos2 under normal growth conditions, probability distribution of the mean of six successive one-step estimates of the diffusion coefficient, *P*(<*D*>_6-step_). Cutoff values of *D* = 1.56 µm^2^/s and *D* = 7.8 µm^2^/s select the 10% slowest and the 10% fastest mean values. (B) Mean square displacement plots, MSD(τ), for the fastest 10% of EF-P–mEos2 trajectories (red circles) and the slowest 10% (black circles). Error estimates are ±1 σ of the MSD values. The slope of the first two data points yields diffusion coefficient estimates of 8.65 µm^2^/s and 0.66 μm^2^/s, respectively. Intercepts are used to estimate dynamic localization errors σ_fast_ and σ_slow_. Download FIG S5, TIF file, 31 MB. Copyright © 2017 Mohapatra et al. 2017 Mohapatra et al. This content is distributed under the terms of the Creative Commons Attribution 4.0 International license.

10.1128/mBio.00300-17.7 FIG S6  Experimental probability distribution of the mean of six successive single-step displacements of EF-P–mEos2 trajectories truncated to 6 steps, *P*(<*r*>_6_) (bold line). Dashed lines are simulated results for two-state models with binding-unbinding kinetics. All simulations use the model parameters *D*_slow_ = 0.2 µm^2^/s (σ_slow_ = 50 nm), *f*_slow_ = 0.3, *D*_fast_ = 4.3 µm^2^/s (σ_fast_ = 75 nm), and *f*_fast_ = 0.7, taken from the best fit to the single-step *P*_EF-P_(*r*) as in Fig. 4A. The ratio τ_free_/τ_bound_ = 7/3 is fixed by the best-fit fractions. The best fit has τ_free_ = 16 ms (and τ_bound_ = 7 ms), with χ_ν_^2^ = 1.4, as shown in Fig. 4. The simulation results shown for τ_free_ = 11 ms (χ_ν_^2^ = 1.95) and 20 ms (χ_ν_^2^ = 2.12) set the error bars on τ_free_. Also shown are simulations for τ_free_ = 0.1 ms (fast exchange limit compared with 2-ms camera frame time) and 100 ms (slow exchange limit), which give worse fits. Download FIG S6, TIF file, 42.1 MB. Copyright © 2017 Mohapatra et al. 2017 Mohapatra et al. This content is distributed under the terms of the Creative Commons Attribution 4.0 International license.

There are about 40,000 translating ribosomes in the average *E. coli* cell volume of ~3 μm^3^, a concentration of ~2.2 × 10^−5^ M. The effective bimolecular rate constant for EF-P binding to translating ribosomes in the living cell can then be estimated as *k*_bimol_ = τ_free_^−1^ [ribosomes]^−1^ ≈ 2.9 × 10^6^ M^−1^ s^−1^. This is ~100 times slower than the diffusion limited rate constant for EF-P–ribosome collisions, *k*_diff_ ≈ 4 × 10^8^ M^−1^ s^−1^. The inefficiency is likely due to the small target site on the ribosome, the small fraction of an elongation cycle during which the E site is open for EF-P binding, and the steric requirements for binding.

Analogous lifetime analysis of the EF-P diffusion data after Cam treatment gave best-fit lifetimes of τ_free_ = 9 ms and τ_bound_ = 7 ms. The ribosome stalling under Cam treatment is presumably essentially permanent (40). There is no active translation that might push EF-P from its binding site as a deacylated tRNA translocates into the E site. This suggests that τ_bound_ ≈ 7 ms is the natural lifetime of an EF-P/ribosome complex independent of whether or not elongation is actually occurring. The cycle time for binding/unbinding events under Cam treatment becomes (τ_bound_ + τ_free_) ≈ 16 ms, shorter than in normal growth because more binding sites are available for the search process and the binding sites presumably remain open.

## DISCUSSION

The crystal structure indicates that EF-P binds between the P and E sites of a translating 70S ribosome, i.e., a binding event requires an empty E site (12). Studies of translation kinetics *in vitro* and *in vivo* found that wild-type EF-P binds to translating ribosomes and alleviates pausing at Pro-Pro motifs (4, 6–8). Those studies further suggested that pausing during a slow peptidyl transfer step may be a prerequisite to EF-P binding. For Pro-Pro motifs, the release of deacylated tRNA from the E site during the elongation round preceding the translational pause presumably renders the E site accessible sufficiently long for free EF-P to find its binding site. The bound EF-P then catalyzes more efficient incorporation of the P-site Pro into the nascent peptide chain. Kinetics data also showed that without the β-lysine modification at Lys-34, binding of EF-P to translating ribosomes diminished 30-fold (4).

Our results *in vivo* are consistent with the binding and mechanistic insights gleaned from the work *in vitro* and add new information about the timescale and frequency of typical EF-P–ribosome interactions. The axial spatial distribution of wild-type EF-P is three-peaked, qualitatively similar to that of the ribosomes (Fig. 2). This shows that EF-P concentrates in the ribosome-rich regions where most translation occurs. Based on the diffusion measurements, about 30% of EF-P copies are associated with translating ribosomes at a given time (Fig. 4A). The translation-halting drug chloramphenicol is known to inhibit peptidyl bond formation and thereby stall translation. Cam treatment increases the fraction of EF-P associated with ribosomes to about 45% (Table 1; Fig. S3A), consistent with the idea that pausing and opening of the E site enable EF-P binding. The diffusion studies after Rif treatment (Table 1; Fig. S3B) show no clear evidence of binding of EF-P to free ribosomal 30S or 50S subunits.

The new data also indicate that there is little or no binding *in vivo* of the mutant form EF-P^K34A^ to 70S ribosomes. The spatial distribution of EF-P^K34A^ is homogenous (Fig. 3B), unlike the three-peaked axial distribution of wild-type EF-P and ribosomes. In addition, the diffusion measurements show no clear evidence of a fraction of the EF-P^K34A^ population that diffuses like the slow, translating ribosomes (Fig. 3C). However, the one-step *P*(*r*) for EF-P^K34A^ is best fit to two populations, with *D*_slow_ = 3.2 µm^2^/s and *D*_fast_ = 9.7 µm^2^/s (Table 1). It is possible that the slower component results from very fast exchange between short-lived EF-P^K34A^/ribosome complexes and freely diffusing EF-P^K34A^.

For wild-type EF-P, two-state analysis of the diffusion data yielded rough estimates of the mean search time for EF-P to find a binding site (τ_free_ ≈ 16 ms) and the mean duration of a typical binding event (τ_bound_ ≈ 7 ms). The mean protein production rate in rapidly growing *E. coli* is ~20 amino acids per ribosome per s. This sets an upper limit of ~50 ms on the average duration of a single elongation cycle, including aminoacyl-tRNA binding, peptide bond formation, and translocation. Our estimate of τ_bound_ is much shorter than the average elongation cycle time.

The detailed nature of these binding events is uncertain. *In vitro*, EF-P associates with translating, 70S ribosomes in a stoichiometric ratio of 1:1 (11). Quantitative interpretation of our results requires an estimate of the EF-P copy number. An early analysis using radioactive labeling and two-dimensional gels found that EF-P is present at about 0.1 copies per ribosome, independent of growth conditions (41). Under our growth conditions, on average there are ~50,000 total 30S copies per cell (30), suggesting only ~5,000 EF-P copies. However, a recent mass spectrometric study found ~20,000 EF-P copies per cell, a substantially larger number (42). Some 80% of 30S copies are incorporated into 70S (translating) ribosomes, indicating 40,000 translating ribosomes under our conditions. The *P*_EF-P_(*r*) data indicate that roughly 30% of the EF-P copies are bound to a translating ribosome at a given moment. Depending on which copy number estimate we use, this implies that a snapshot would show 1,500 to 6,000 EF-P/ribosome complexes at a given moment in time.

For either copy number estimate, there are evidently too few Pro-Pro motifs in the single-cell transcriptome of *E. coli* to account for so many simultaneous EF-P/ribosome-binding events. First, we use the most recent mass spectrometric data (42) and known gene sequences to estimate the fraction of Pro-Pro motifs within the set of transcripts that have been translated to produce the detailed set of proteins present per *E. coli* cell. Then, we multiply the fraction of Pro-Pro motifs by the mean number of mRNA codons present in one cell to estimate the total number of Pro-Pro motifs present in the single-cell transcriptome at a given time. Our estimate assumes similar degradation rates for all proteins, so that the proteome provides a representative sample of the distribution of translated codon motifs.

A recent mass spectrometric study found 1,611 proteins with abundances greater than 20 per cell for *E. coli* strain MG1655 growing exponentially in glucose minimal medium (42). The 1,611-member sample had an average of 260 codons per protein. The total number of such protein copies was 4.8 × 10^6^ per cell. Those proteins comprise 1.3 × 10^9^ total amino acids, whose corresponding codons we assume to form a representative sample of the mRNA codons that are transcribed to make the proteome of one cell. For each of the 1,611 genes in that codon sample, we multiplied the number of Pro-Pro motifs by the corresponding protein copy number to obtain the total number of Pro-Pro motifs in the sample, which is 6.6 × 10^5^. Less than 3% of the total number of Pro-Pro motifs comprise Pro-Pro-Pro triplets. The fractional occurrence of Pro-Pro motifs that were translated to form the representative protein sample is roughly 6.6 × 10^5^/1.3 × 10^9^ = 5.3 × 10^−4^ ≈ 0.05%.

How many total codons comprise the transcriptome (mRNA content) of the typical *E. coli* cell in exponential growth? The classic literature estimate for total mRNA copy number is 1,200 (43) for the B/r strain growing in minimal medium at 37°C (doubling time of 40 min, similar to our VH1000 strain in EZ rich defined medium [EZRDM] at 30°C). We take 2,000 mRNA copies/cell as an estimate for our conditions. The total number of Pro-Pro motifs present at a given moment in one *E. coli* cell is then ~2,000 mRNA copies × 260 codons/mRNA × 5.3 × 10^−4^ ≈ 280 Pro-Pro/cell. That is 5 times smaller than the lower estimate of 1,500 simultaneous EF-P/ribosome-binding events and 20 times smaller than the higher estimate of 6,000 simultaneous binding events.

Thus, it seems unlikely that the rare events in which EF-P is relieving a pause at a Pro-Pro motif cause a significant fraction of the binding events that we have detected. Of course, there are other motifs that cause pauses that are rescued by EF-P, but these are a minority of the pausing events detected in ribosome profiling studies on *efp* mutant strains. Instead, it seems plausible that the typical EF-P/ribosome-binding event detected in our study is a kind of “housekeeping” event involving short-lived open E sites that may arise routinely when a wide variety of codons are present at the P site. When an EF-P finds an open E site, it typically binds very briefly (τ_bound_ ≈ 7 ms) before dissociating and beginning another search. The search lasts for only a short time (τ_free_ ≈ 16 ms). If the typical timescale of a searching/binding cycle is (τ_free_ + τ_bound_) ≈ 23 ms, then each EF-P copy would carry out some 40 cycles per s.

According to these estimates, the 5,000 to 20,000 EF-P copies combined would carry out ~200,000 to 800,000 “ribosome E-site interrogations” per s. On average, each of the 40,000 translating ribosomes would experience an EF-P binding event about 5 to 20 times per s. The 40,000 ribosomes translating at 20 amino acids (aa) per s carry out a total of 800,000 elongation cycles per s, which is comparable to the rate of interrogation by EF-P. This means that roughly 25 to 100% of all translation cycles leave the E site open for a sufficiently long period to enable EF-P to bind. Definitive determination of the EF-P copy number would sharpen that estimate. Regardless, the typical binding time of ~7 ms remains short compared with the overall elongation cycle time of ~50 ms. Our data do not constrain the binding time during those evidently rare events in which EF-P binds to a ribosome that is stalled at a Pro-Pro motif. The Pro-Pro rescue time could be similar to or substantially longer than 7 ms. However, we can estimate that each of the 40,000 translating ribosomes experiences at least 5 and perhaps as many as 20 of the brief EF-P visits per s. Evidently, the cell contains enough EF-P copies to ensure that long translational pausing events, such as those that might occur at Pro-Pro, would catch an EF-P on a timescale of ~200 ms or less.

It is possible that most of these brief EF-P binding events are nonfunctional, but essentially harmless, due to their short duration compared with an elongation cycle. Such frequent visits may at least serve the purpose of expediting the relief of pauses at Pro-Pro duets when they do arise. The stalled ribosome need not wait long for an EF-P arrival. Alternatively, the typical EF-P binding event help to alleviate brief pauses that are not yet detectable in ribosome profiling studies, because they are too short to be observed as a buildup of ribosome density. Or, these events may serve some different purpose.

Finally, the occurrence of an EF-P binding event during many or most elongation cycles is consistent with recent single-molecule translation studies implying that the deacylated tRNA dissociates from the E site after or during the translocation step (20–22). Our *in vivo* data are not consistent with a concerted mechanism of tRNA progression through the A, P, and E sites, i.e., a mechanism in which the E site is never completely empty. Our results do not speak to the longstanding issue of whether or not dissociation of the deacylated tRNA from the E site occurs before or after binding of a new aa-tRNA at the A site (20–25).

## MATERIALS AND METHODS

### Strain construction.

All experiments were performed on *E. coli* of background strain VH1000. Wild-type and modified strains are described in Table S1 in the supplemental material. For imaging EF-P, we constructed an *E. coli* strain that expresses EF-P translationally fused with the photoswitchable protein mEos2 at the C terminus.

The oligonucleotides used for this work are listed in Table S1. The mEos2-encoding gene was first amplified with primers *meos_F* and *meos_R* from plasmid pUC57-mEos2 and then subcloned into the plasmid pREST-B between BamHI and EcoRI sites to generate a tandemly placed mEos2 open reading frame (ORF) and kanamycin resistance (Kan^r^) cassette separated by a linker containing an independent ribosome-binding site for Kan^r^. A linear DNA containing the mEos2-Kan^r^ cassette was subsequently amplified with primers *efpmeos_F* and *efpmeos_R*, which contained sequences homologous to immediate up- and downstream regions of the stop codon of the *efp* gene, present at the b4147 locus in the genome of *E. coli* MG1655 (ECK4141). Next, the stop codon of the *efp* gene on the chromosome of *E. coli* MG1655 was replaced with the mEos2-Kan^r^ cassette using λ-Red recombineering (44–46). The resulting recombinants with EF-P–mEos2 fusion were selected by screening for kanamycin resistance and further verified by DNA sequencing. One of the successful recombinants was named XG501 (MG1655 *efp-mEos2 Kan*), which was subjected to growth analysis and further strain preparation. The *efp-mEos2 Kan* fusion was then moved into a clean VH1000 background by P1 transduction, resulting in strain SM1.

The *efp-mEos2* cassette was PCR amplified by forward primer EM1, introducing BamHI, and reverse primer EM2, introducing HindIII. The PCR-amplified product was ligated into pASK-IBA3plus (Invitrogen, Carlsbad, CA) double digested with BamHI and HindIII. VH1000 cells were transformed with the resulting plasmid (pSM1), and transformants were selected on ampicillin-containing plates. This strain (SM4) was used for control experiments for imaging EF-P–mEos2 being expressed from the plasmid upon induction.

PCR amplification of pSM1 using oligonucleotides Mut1 and Mut2 and the commercially available Phusion site-directed mutagenesis kit was used to mutagenize the Lys-34 residue to Ala. This PCR-amplified product was further ligated to generate pSM2. VH1000 cells were transformed with pSM2, and transformants (SM8) were selected on ampicillin-containing plates.

The doubling time of strain SM1 in bulk EZ rich defined medium (EZRDM) at 30°C (47) is 60 ± 3 min (Table S1B), a factor of 1.3 longer than the doubling time of 45 min of the wild-type VH1000 under the same conditions (30). For the plasmid-containing strains SM4 and SM8, doubling times without induction were 51 ± 1 min and 50 ± 6 min, respectively (Table S1B).

### Cell growth and preparation for imaging.

Bulk cultures from frozen glycerol stock solution and subcultures for imaging were grown at 30°C with continuous shaking in EZRDM, which is a morpholinepropanesulfonic acid (MOPS)-buffered solution with supplemental metal ions (M2130; Teknova), glucose (2 mg/ml), supplemental amino acids and vitamins (M2104; Teknova), nitrogenous bases (M2103; Teknova), 1.32 mM K_2_HPO4, and 76 mM NaCl.

The strains that include the plasmid expressing EF-P–mEos2 or EF-P^K34A^–mEos2 were grown with addition of 100 µg/ml ampicillin. When cells had grown to mid-log phase, anhydrotetracycline was added to a final concentration of 45 nM to induce the expression of the desired protein. After 5 min of induction, the cells were centrifuged and resuspended in fresh EZRDM with 100 µg/ml ampicillin to remove the inducer. The cells were then incubated again in growth medium for 30 to 45 min at 30°C to enable maturation of the fluorescent protein prior to imaging.

Rifampin and chloramphenicol stock solutions were prepared by dissolving 10 and 15 mg of the drugs in 0.5 ml of ethanol, respectively. Stock solutions of the drugs were added to the mid-log cell culture to attain final concentrations of 300 µg/ml and 200 µg/ml, respectively. For imaging of EF-P under rifampin and chloramphenicol treatment, the cultures were shaken for 3 h and 30 min, respectively, before plating and imaging.

### Superresolution imaging of live *E. coli* cells.

Fluorophore-labeled cells were grown overnight with shaking at 30°C in EZRDM. Subcultures were made by diluting the stationary-phase culture at least 1:100 into 2 ml of fresh EZRDM. Subcultures were grown to exponential phase (optical density [OD] = 0.2 to 0.6 at 600 nm), upon which the culture was placed in CoverWell perfusion chamber gaskets (Invitrogen, Carlsbad, CA) on a polylysine-coated coverslip. The volume of the closed chamber is 150 µl. We allowed ~2 min for the cells to adhere to the coverslip and then replaced the liquid in the chamber with fresh, aerated medium to rinse away the nonadhered cells. Live-cell imaging was carried out at 30°C for no longer than 30 min after plating. During that time, cells continued to grow.

Cells were imaged using an Eclipse Ti inverted microscope (Nikon, Melville, NY) equipped with an oil immersion objective (CFI Plan Apo Lambda DM 100× oil, 1.45 numerical aperture [NA]; Nikon Instruments), a 1.5× tube lens, and the Perfect focus system (Nikon, Melville, NY). For time-lapse imaging, fast shutters (Uniblitz LS2; Vincent Associates, Rochester, NY) were used to synchronize illumination and image acquisition. Images were recorded by a fast back-illuminated electron-multiplying charge-coupled device (EMCCD) camera with 128 by 128 pixels of 24 by 24 μm each (iXon DV-860; Andor Technology, South Windsor, CT). Each pixel corresponds to 160 by 160 nm^2^ at the sample (overall magnification, ×150). All data were collected at a frame rate of 485.4 Hz, with exposure time within each frame of 2 ms. The mEos2 was photoactivated with a 405-nm diode laser (CrystaLaser, Reno, NV) and subsequently imaged with a 561-nm laser (Sapphire 561 CW lasers; Coherent, Bloomfield, CT). The 405-nm power density at the sample was 15 to 25 W/cm^2^. The photoactivation laser remained on throughout imaging. Power density of the 561-nm laser was kept at ~8 kW/cm^2^. The probe laser was on continuously in the 2-ms/frame tracking experiments. To minimize the phototoxic effect of the laser, we collected data for <25 s per cell. The mEos2 emission was collected through a 617/73 band-pass filter (bright line, 617/73 to 25; Semrock, Rochester, NY).

### Single-molecule image analysis.

Images were analyzed using a Matlab graphical user interface (GUI) developed in our lab (48). Images were smoothed and filtered to obtain a zero-based image. Bright spots were located with pixel-level accuracy by a peak-finding algorithm that finds local intensity maxima within an image. A user-defined intensity threshold was used as the minimum brightness of a pixel from single-molecule trajectories. It is carefully set by the user so that it will not be so high as to cut a long trajectory short or so low as to include background noise.

Centroids of the bright spots were calculated from a 7- by 7-pixel square centered on the local maxima determined by the peak-finding algorithm. As the images are asymmetrically blurred due to diffusion during the frame time, we calculate the centroid of the bright spots instead of using Gaussian fitting. This centroid analysis is fast and easily implemented for analysis of Monte Carlo modeling results as well. For single-molecule tracking analysis, the (*x*, *y*) positions of the centroid are stored and connected into trajectories using a modified Matlab version of the tracking program written by Crocker and Grier (49).

In order to generate the axial distribution of molecules from several cells, the camera-based coordinates are reoriented so that the *x* axis corresponds to the long cell axis. The axial positions are scaled from −0.5 to 0.5 using the cell length determined by MicrobeTracker (50) from phase-contrast images.

### Analysis of diffusive behavior.

Details of mean square displacement plots, trajectory simulations, two-state modeling of *P*_(*r*)_ and *P*(<*r*>_6-step_) distributions, and estimation of uncertainties in fitting parameters are provided in the supplemental material.

10.1128/mBio.00300-17.8 FIG S7  Reduced chi-square values χ_ν_^2^ for fits to *P*_EF-P_(*r*) using different parameter sets. Three slices through the three-dimensional grid of χ_ν_^2^ values (with fitting parameters *D*_slow_, *D*_fast_, and *f*_slow_) obtained in modeling the experimental distribution of one-step displacements in Fig. 4A. (A) Slice through the plane with *D*_slow_ = 0.2 μm/s; *D*_fast_ and *f*_slow_ vary. (B) Slice through the plane with *D*_fast_ = 4.3 μm^2^/s; *D*_slow_ and *f*_slow_ vary. (C) Slice through the plane with *f*_slow_ = 0.30; *D*_slow_ and *D*_fast_ vary. Regions outlined in blue have χ_ν_^2^ ≤ 1.5 and produce qualitatively poorer fits than those with χ_ν_^2^ ≈ 1. Those regions were used for error estimates on best-fit parameters. Download FIG S7, TIF file, 2.1 MB. Copyright © 2017 Mohapatra et al. 2017 Mohapatra et al. This content is distributed under the terms of the Creative Commons Attribution 4.0 International license.

10.1128/mBio.00300-17.10 TABLE S2  Summary of range of fitting searches for *P*_(*r*)_ for different species and imaging conditions. Download TABLE S2, DOCX file, 0.01 MB. Copyright © 2017 Mohapatra et al. 2017 Mohapatra et al. This content is distributed under the terms of the Creative Commons Attribution 4.0 International license.
