# Supplementary material for: Spatial Distribution and Ribosome-Binding Dynamics of EF-P in Live Escherichia coli
Source: mBio. 2017 Jun 6;8(3):e00300-17. doi: 10.1128/mBio.00300-17 (PMC5461404; doi:10.1128/mBio.00300-17)
Supplement: TABLE S1 [file mbo003173332st1.docx]

**Table S1A.** Oligonucleotides used in construction of strains described in the main text.

| Oligonucleotide | Sequence 5′- 3 ′ |
| --- | --- |
| *meos_F* | CGC GGATCC ATGTCTGCTATTAAACCGG |
| *meos_R* | CCG GAATCC TTAGCGACGAGCATTATCCG |
| *efpmeos_F* | AGTGGATACCCGCTCTGGTGAATACGTCTCTCGCGTGAAGTCTGCTATTAAACCGGATATGAAGA |
| *efpmeos_R* | ATAAGTGATGGTGCAGCCTGCAGGCCGCACCACAACCGCATTAGAAAAACTCATCGAGCATC |
|  |  |
| *EM1*  *EM2*  *Mut1*  *Mut2* | GATCGGATCCATTTCAGAGGGCCTTATGGC  GATCAAGCTTTTAGCGACGAGCATTATCCGG  AAAACCGGGT**GCA**GGCCAG  ACGAATTCACTCGCTTCAACCG |

**Table S1B.** Strains used for imaging and doubling times at 30°C in EZ rich, defined medium (EZRDM).

| **Strain name** | **Description** | **Doubling time  (min)** |
| --- | --- | --- |
| SM1 | EF-P–mEos2 (chromosome) | 60 ± 3 |
| SM4 | EF-P–mEos2 (plasmid) | 51± 1 |
| SM8 | EF-P^K34A^ –mEos2 (plasmid) | 50 ± 6 |
| MSG196 | Ribosome S2–mEos2 (chromosome) | 51± 3 |
| VH1000 | Wild type | 45 ± 1(1) |
