# Supplementary material for: Spatial Distribution and Ribosome-Binding Dynamics of EF-P in Live Escherichia coli
Source: mBio. 2017 Jun 6;8(3):e00300-17. doi: 10.1128/mBio.00300-17 (PMC5461404; doi:10.1128/mBio.00300-17)
Supplement: TEXT S1 [file mbo003173332s1.docx]

**Supplemental Material**

***E. coli* strains and oligonucleotides used in this work**

See Table S1.

**Axial distributions under drug treatments**

Figures S1A and S1B show 2D heat maps and 1D axial projections of the spatial distributions of EF-P–mEos2 and ribosomes (30S–mEos2 labeling) following treatment with chloramphenicol (Cam). Figures S1C and D show analogous distributions following treatment with rifampicin (Rif).

**Analysis of diffusive behavior**

***Mean-square displacement plots MSD*(τ)**

The MSD as a function of lag time * *is given by $MSD\left( \tau\right)= <{(\boldsymbol{r}\left( t+\tau\right)-\boldsymbol{r}\left( t \right))}^{2}>$, where ***r***(*t*) is the two-dimensional location of the particle at time *t*,  is the lag time, and the average is taken over all times *t* and over many trajectories. MSD(τ) plots for 30S-labeled ribosomes and wild-type EF-P in normal growth conditions are shown in Fig. S4A. Plots for EF-P^K34A^ in normal growth and for wild-type EF-P after Rif treatment are shown in Fig. S4B.

The slope of the first two points of an MSD(τ) plot provides a first estimate of the true mean diffusion coefficient: *D* = slope/4. The MSD slope takes account of the dynamic localization error , but does not account for confinement effects. Even for 6 x 2 ms = 12 ms long trajectories, for rapidly diffusing species with *D* ~ 5 μm^2^-s^-1^ confinement restricts diffusive trajectories and causes curvature of the MSD plot. This makes the diffusion coefficient from the two-point slope a lower bound on the true *D.* The trajectory analysis presented below is more accurate.

***Estimation of dynamic localization error σ_fast_ and σ_slow_ for fast and slow EF-P molecules***

Localization error in the single-step *P*(*r*) distributions arises from the point-spread function (PSF) of the microscope, the finite number of photons detected per camera frame, and blurring of the images due to diffusive motion during each 2 ms camera frame (2, 3). While ribosomes and ribosome-bound EF-P copies experience little diffusive blurring, the rapidly moving free EF-P copies are blurred substantially. We reasoned that model Monte Carlo diffusive trajectories should involve larger “dynamic localization error” for fast EF-P copies than for slow EF-P copies.

This problem has been addressed in detail by Michalet (2). Suppose the best fit to the first two experimental points of a two dimensional mean-square displacement plot is given by the equation MSD(τ) = *a* + *b*τ, with *b* the slope and *a* the extrapolated intercept at lag time  = 0. Then the best estimate of the diffusion coefficient is *D* = *b*/4 and the best estimate of the dynamic localization error is  = ½ (*a* + 4*Dt*_E_/3)^1/2^, where *t*_E_ is the exposure time per camera frame. From the MSD plots for the total populations of EF-P and of ribosomes (Fig. S4A), we estimate that the diffusion coefficient of the free EF-P copies should be about 4 μm^2^-s^-1^ and that of the ribosome-bound copies should be about 0.5 μm^2^-s^-1^. If so, the root-mean-square displacement in two dimensions of a fast EF-P copy during the 2-ms camera frame is ~200 nm, substantially larger than typical static localization errors in live *E. coli* single-molecule studies of slowly moving species labeled with fluorescent proteins. The rms displacement of a slow EF-P copy is only ~60 nm, comparable to typical static localization errors.

To estimate the dynamic localization errors *_fast_* and *_slow_* for EF-P, we used six-step trajectories to form the distribution of the mean of six one-step estimates of *D*:
${<D>}_{6-step} = \frac{1}{24\tau}\sum_{i=1}^{6} \sqrt{({x_{i+1}-x_{i})}^{2}+({y_{i+1}-y_{i})}^{2}}$, where (*x_i+_*_1_*, y_i+_*_1_) and (*x_i_, y_i_*) are the coordinates of final and initial positions of each step. This distribution is shown in Fig. S5A. Separate MSD plots for the slowest 10% of trajectories and the fastest 10% of trajectories are shown in Fig. S5B. The slope of the linear fit to the first two data points gives nominal slow and fast diffusion coefficients of 0.7 and 8.6 μm^2^-s^-1^, respectively. These results are combined with the intercepts to give the estimates σ_slow_ ~ 40 nm and σ_fast_ ~ 86 nm. Because the analysis used the slowest and fastest copies, we chose σ_slow_ = 50 nm and σ_fast_ = 75 nm when simulating the diffusive trajectories used to fit *P_EF-P_*(*r*) in Fig. 3A. A similar method was used for the ribosome trajectories. It yielded the estimates σ_slow_ = 40 nm and σ_fast_ = 75 nm, which were used in fitting *P_ribo_*(*r*) in Fig. 3B.

**Monte Carlo simulations of diffusive trajectories**

Many previous studies of single-molecule diffusion fit the experimental *P*(*r*) distribution to a sum of analytical functions, with each component describing diffusion of the species in free space (4-7). However, molecules diffusing rapidly in the *E. coli* cytoplasm suffer from confinement due to the spherocylindrical cell boundaries, a problem for which there is no analytical solution. Therefore, we simulated the behavior of each diffusive component from a large number of random walk trajectories that incorporate the dynamic localization error  and confinement effects within a model spherocylinder that mimics the dimensions of a typical *E. coli* cell in our growth conditions (tip-to-tip cell length = 4 μm and cell diameter = 0.9 μm). Each set of simulations models one diffusive state, with values of D and  fixed. At t = 0, 15,000 particles were randomly distributed within the cell volume. Each particle undergoes a random walk independent of other particle positions. To model each 2-ms camera image, three-dimensional microtrajectories (1000 steps of 2 μs each) were generated. At each time step, each particle chooses a displacement in each of three Cartesian directions. These displacements are chosen from a Gaussian distribution whose standard deviation corresponds to the state’s three-dimensional diffusion coefficient D. In the rare event that a particle attempts to step outside of the cell boundaries, the displacement for that microstep is taken to be zero. The location of each particle during each camera frame is obtained as the centroid of the model microtrajectories in order to mimic the analysis procedure used for the experimental images. The appropriate dynamic localization error  was then applied to each centroid location in both *x* and *y* coordinates by sampling a Gaussian distribution with standard deviation  By adding the error to the centroid position we obtain the model “measured” location for each 2 ms camera frame. The *x* and *y* coordinates of each measured location are stored for further analysis. For the next model camera frame, each particle continues to make microsteps in 3D starting from the endpoint of the previous camera frame. Model trajectories for EF-P and for ribosomes use the appropriate estimated value of σ_slow_ or σ_fast_, determined as described above.

By connecting the sequence of “measured” simulation locations over seven frames, we form 15,000 model trajectories for each relevant value of *D*. These trajectories are used to compute model-based, numerical one-step probability distributions *P_model_*(*r*;*D*) that are the model functions for the least-squares analysis of the corresponding experimental distributions.

***Fitting of ribosomal distribution P_ribo_(r) to a static, two-state model***

As shown previously (5), single-molecule methods with the 30S ribosomal subunit labeled can resolve two diffusive states of ribosome in live *E. coli*: slowly diffusing, translating 70S ribosomes (likely present as polysomes) and more rapidly diffusing, free 30S subunits. Ideally, if the transition time scale between these two states is much longer than the experimental observation time scale of single camera frames (2 ms), the magnitude of displacements for individual trajectory steps should indicate the diffusive state of the molecule. Nearly 15,000 experimental trajectories of 30S-labeled ribosomes that lasted for 6 steps or longer were selected for analysis. Longer trajectories were truncated to 6 steps. The 6-step trajectories were then sliced into individual steps. The single-step displacements
 $r_{i}=\sqrt{({x_{i+1}-x_{i})}^{2}+({y_{i+1}-y_{i})}^{2}}$were pooled to form the distribution *P_ribo_*(*r*) in Fig. 3B.

We judge the goodness of fit by evaluating the reduced chi-square statistic:. Here *j* labels the *N* bins in the (unnormalized) *P*(*r*) and *P_model_*(*r*) histograms, *h_j_* is the number of experimental counts in bin *j*, *y_j_* is the number of counts in bin *j* of the simulated *P_model_*(*D_i_*) histogram, is the variance of the value in bin *j*, and α is the number of fitted parameters. We take = *h_j_* as the estimate of the variance, assuming Poisson statistics. A good fit to an adequately flexible *analytical* model function should have __^2^ ~ 1. Since our model functions are numerical, there is noise in both the experimental and simulated histograms. In some cases, the number of experimental and model input trajectories are comparable, in which case a good fit could have __^2^ ~ 2.

As judged by the reduced chi-square statistic __^2^, the distribution *P_ribo_*(*r*) is poorly fit by a one-state model, *i.e.*, to a single numerical function *P_model_*(*r*;*D*). Next we modeled *P_ribo_*(*r*) as a sum of two static (non-exchanging) populations: *P_model_*(*r*) = *f_slow_P*(*r*;*D_slow_*) + (1 – *f_slow_*)*P*(*r*;*D_fast_*). The appropriate values of *_fast_* and *_slow_* were used for each component. The least-squares fitting procedure involved a numerical search for the lowest value of χ_ν_^2^ on a 3D grid of combinations of the three independent adjustable parameters (*D_fast_*, *D_slow_*, *f_slow_*). The best fit to this model function yielded *f_slow_* = (1 – *f_fast_*) = 0.65, *D_slow_* = 0.2 µm^2^/s (presumably the translating 70S ribosomes), *f_fast_* = 0.35, and *D_fast_* = 0.8 µm^2^/s (presumably the free 30S subunits). The minimum goodness-of-fit parameter was χ_ν_^2^ = 2.3. Due to the large number of ribosome trajectories (15,000), we used the same number of model trajectories in the simulation. Both experimental data and the numerical model function have noise. The noise in the model function contributes to the value of χ_ν_^2^, so a value near one is not expected. The best fit is qualitatively good, as seen in Fig. 3B. Additionally, the best-fit combination of parameters gives the weighted average
<*D_ribo_*> = *f_slow_D_slow_* + *f_fast_D_fast_* = 0.41 μm^2^-s^-1^. This agrees reasonably well with the mean value <*D_ribo_*> = 0.49 μm^2^-s^-1^ from the initial slope of the MSD plot (Fig. S4A). We conclude that the static, two-state model is reasonable for fitting the *P_ribo_*(*r*) distribution.

For comparison, we also simulated *P_ribo_*(*r*) using a static, two-state model in which both the fast and slow constituent populations have the same $\sigma$ = 57 nm, as estimated from the MSD including all ribosome trajectories. The resulting χ_ν_^2^ were always very large (> 35).

***Fitting of EF-P distribution P_EF-P_(r) to a static, two-state model***

The distribution of one-step displacements *P_EF-P_*(*r*) in Fig. 4A was generated from 1000 six-step trajectories for 2-ms camera frames. We first attempted to fit the distribution to a single diffusive state using 15,000 simulated EF-P trajectories of varying *D* and σ = 61 nm, obtained from the intercept of MSD(τ) plots for all EF-P trajectories (Fig. S4A). However, the best one-state fit had *D* = 3.1 μm^2^-s^-1^ and an unacceptably large value χ_ν_^2^ = 5.0.

Then we tested two-state models of EF-P with no exchange between states on the 2-ms timescale. The two states were a slowly diffusing, ribosome-bound state (*D_slow_*) and a rapidly diffusing, free state (*D_fast_*), with population fractions *f_slow_*and *f_fast_*. Appropriate values of *_slow_* and *_fast_* were used, as described above. Trial and error showed that the large measurement error precludes accurate determination of *D_slow_*. In most of the fitting effort, we therefore constrained *D_slow_* = 0.2 μm^2^-s^-1^ to match that of the best-fit slow component of *P_ribo_*(*r*) and optimized *f_slow_* and *D_fast_.* For each combination of input parameters selected from a 2D grid of values, we generated some 15,000 individual simulated trajectories, yielding a numerical model function
*P_model_*(*r*) = *f_slow_P*(*r*;*D_slow_*) + *f_fast_P*(*r*;*D_fast_*). The best fit was obtained for *f_slow_*= 0.30,
*D_fast_* = 4.3 μm^2^-s^-1^, and *f_fast_* = 0.70. The corresponding goodness-of-fit parameter was χ_ν_^2^ = 1.0, indicating a good fit. We are now using 15,000 model trajectories to generate each model function, while the experimental *P_EF-P_*(*r*) distribution is derived from only 1000 trajectories. The noise in the data is substantially larger than that in the numerical model function, so a value of χ_ν_^2^ near one should be expected if the model function is adequate. This fit is compared with the data in Fig. 4A. We identify the slowly diffusing EF-P fraction as the copies bound to translating 70S ribosomes and the rapidly diffusing fraction as free EF-P, consistent with our original estimate of the diffusion coefficient for free copies. These parameters give a mean diffusion coefficient of *<D>* = 3.1 μm^2^-s^-1^, quite similar to *<D>* = 3.4 μm^2^-s^-1^ obtained from the MSD(τ) plot for all EF-P trajectories (Fig. S4A).

Upon optimizing *D_slow_*, *f_slow_*, *D_fast_* and *f_fast_*without any constraints on *D_slow_*, the best fit was obtained for *D_slow_*= 0.5 μm^2^-s^-1^, *f_slow_*= 0.35, *D_fast_* = 4.8 μm^2^-s^-1^, and *f_fast_* = (1 – *f_slow_*) = 0.65 with χ_ν_^2^ = 0.78 (slightly better than the best constrained fit). Thus the fractions and diffusion coefficients obtained from an unconstrained fit are quite similar to those from the constrained fit using *D_slow_* = 0.2 μm^2^-s^-1^.

To estimate the uncertainty in the model parameters, we examined the 3D grid of χ_ν_^2^ values generated from the unconstrained fits that varied all three parameters *D_fast_*, *f_slow_*, and *D_slow_*. Three two-dimensional planes passing through the values *D_fast_* = 4.3 μm^2^-s^-1^, *f_slow_* = 0.30, and
*D_slow_* = 0.2 μm^2^-s^-1^ are shown in Fig. S7. We judged the fits to be qualitatively poor whenever the value of χ_ν_^2^ exceeded 1.5, which is 0.5 units larger than the value at our preferred parameter combination. These regions are surrounded by the blue boundaries in the figure. Our best estimates with error bars are *f_slow_*= 0.30 ± 0.10, *f_fast_* = 0.70 ± 0.10, and *D_fast_* = 4.3 ± 1.0 μm^2^-s^-1^. The superimposed heat map colors shows that *f_slow_* and *D_fast_* are positively correlated, *f_slow_* and *D_slow_* are positively correlated, and *D_slow_* and *D_fast_* are essentially uncorrelated. Again, the value of *D_slow_* is not well determined due to the measurement error; reasonable fits can be obtained for *D_slow_* in the range 0–1 μm^2^-s^-1^.

Analogous fitting procedures were carried out for the *P*(*r*) data for EF-P^K34A^ expressed from a plasmid and for wild-type EF-P after drug treatment with Cam and Rif. A summary of the fitting results is provided in Table S2.

**Dynamics of EF-P association with ribosomes**

The fit to the single-step *P_EF-P_*(*r*) distribution assumed two static populations that persist on the 2-ms timescale of a single camera frame. Next we asked whether six-step trajectories (12 ms total duration) can provide information about the timescale of possible transitions between the slow, ribosome-associated state and the fast, freely diffusing state of EF-P. Accordingly, for 859 six-step trajectories of wild type EF-P–mEos2, we generated the distribution *P_EF-P_*(<*r*>_6-step_), the mean displacement of the six steps: ${<r>}_{6-step}= \frac{1}{6}\sum_{i=1}^{6} \sqrt{({x_{i+1}-x_{i})}^{2}+({y_{i+1}-y_{i})}^{2}}$). This is shown in Fig. 5. Longer trajectories are truncated at six steps. Comparing Figs. 4A and 5, we see that *P_EF-P_*(<*r*>_6-step_) is substantially narrower than *P_EF-P_*(*r*) due to the averaging of six displacements, which decreases the relative contribution of measurement error.

We simulated *P_model_*(<*r*>_6-step_) distributions for static populations by averaging the displacements from six-step simulated trajectories that included confinement and measurement error. If the EF-P molecules undergo transitions only rarely during a 12-ms trajectory, then the experimental *P*(<*r*>_6-step_) should match the *P_model_*(<*r*>_6-step_) using the same input parameters that gave the best-fit *P_model_*(*r*) distribution. We simulated 15,000 six-step trajectories,
30% with *D_slow_* = 0.2 µm^2^/s (4500 trajectories) and 70% with *D_fast_* = 4.3 µm^2^/s
(10,500 trajectories), in accord with the parameters of the best-fit model of the *P­_EF-P_*(*r*) distribution of Fig. 4A. *P_model_*(<*r*>_6-step_) for these static populations exhibits two partially resolved peaks (Fig. 5), unlike the experimental distribution. This suggests that the wild type EF-P indeed undergoes transitions during the six-step, 12-ms trajectories.

In order to estimate the lifetime of the two diffusive states, we introduced two-state, binding/unbinding kinetics into our simulations, following Das *et al* (8, 9). Particles initially occupy either of the two states randomly in space in proportion to the population fractions dictated by their lifetimes. When a particle is in a free or bound state, it diffuses with a mean diffusion coefficient of *D_fast_* or *D_slow_*, respectively. It is assumed that the transitions occur instantaneously between two 2-μs long microsteps. The parameters *f_slow_*, *D_slow_*, *f_fast_*, and *D_fast_* were fixed at their preferred values. There are two lifetimes in the problem, τ_free_ (mean search time for EF-P in the freely diffusing state) and τ_bound_ (mean lifetime of EF-P while ribosome-bound). Their ratio τ_free_/τ_bound_ = 7/3 is fixed by the ratio of best-fit population fractions, so there is only one adjustable parameter.

We generated large sets of six-step simulated trajectories with *D_slow_* = 0.2 µm^2^/s, *D_fast_* = 4.3 µm^2^/s and τ_free_/τ_bound_ = 7/3. While keeping τ_free_/τ_bound_ constant, we varied τ_free_ from 0.1 ms to 100, calculating 15,000 trajectories in each case. The best fit to the experimental *P_EF-P_*(<*r*>_6-step_) was obtained for τ_free_ = 16 ms and τ_bound_ = 7 ms, with χ_ν_^2^ = 1.4. This fit is compared with experiment in Fig. 5. Additional calculated distributions *P_model_*(<*r*>_6-step_) for different assumed lifetimes are compared with experiment in Fig. S6. Based on the range of τ_free_ that yields a value of χ_ν_^2^ less than 2.0 (0.6 units larger than optimal), we take τ_free_ = 16 ± 5 ms and τ_bound_ = 7 ± 3 ms as our best estimates of the average lifetime of free EF-P and of EF-P bound to ribosomes, respectively. For χ_ν_^2^ = 2.0 or larger, the fits are qualitatively poorer (Fig. S6). Our best estimate of the typical timescale for an EF-P/ribosome binding/unbinding cycle becomes (τ_bound_ + τ_free_) ~ (23 ± 8) ms.

**Robustness of fitting procedure**

We simulated numerical *P*(*r*) distributions from model trajectories using known input values of the two-state model parameters *f_slow_*, *D_slow_*, *f_fast_*, and *D_fast_* and then tested how well our procedures recover the correct values. This tests the robustness of the procedure for various parameter combinations under the assumption that a two-state model without transitions
on a 2-ms timescale is correct. For example, to mimic the experimental *P_EF-P_*(*r*), we simulated 300 trajectories with *D_slow_* = 0.2 µm^2^/s and σ_slow_ = 50 nm and 700 trajectories with
*D_fast_* = 4.3 µm^2^-s^-1^ and σ_fast_ = 75 nm and combined them to generate a normalized *P_model_*(*r*) to mimic the experimental *P_EF-P_*(*r*). By constraining the model data and experimental data to include the same number of trajectories, they should also have similar noise levels.

Each synthetic model data set is then subjected to the same fitting procedure we used for the experimental data. We repeated the numerical experiment 20 times, producing 20 best fits to the 20 synthetic model data sets. For each fitting parameter, the 20 best-fit are used to produce a mean and standard deviation. The same procedure was carried out for all the cases reported in our study. The table below depicts the reproducibility of the fits for each case. Given that a two-state system without exchange on the 2-ms timescale produced the data, the procedure recovers all parameters to good precision (±10% or better) with the exception of the slowest diffusion coefficients
(±50––100%).

***Closing remarks on data analysis***

It should be clear from the two-state decomposition of the single-step *P_EF-P_*(*r*) distribution (Fig. 3) that the present study approaches the limit of time-resolution of single-molecule tracking methods using fluorescent protein labels. The short frame time of 2 ms is dictated by the need to capture both fast and slow molecules in the same movie. However, the significant measurement error associated with such short observation times blends the apparent fast motion of free EF-P and the apparent slow motion of ribosome-bound EF-P together. They can barely be deconvolved, and the slow diffusion coefficient is only poorly determined. In contrast, the fast and slow fractions are returned with reasonable accuracy, which is important for the main biological conclusions of the Discussion. Finally, it was fortuitous that the bound ↔ free transition times fall within the range that can be unraveled in the short, 6 x 2 ms = 12 ms trajectories that are available experimentally (Fig. 5).

**Supplemental Figure Captions**

**Figure S1.** A) *Top:* Localization probability density heat map of 2220 EF-P–mEos2 copies imaged at 2 ms/frame in different cells of length 2.5–3.5 µm after 30 min of chloramphenicol treatment. Each location is placed on a common scale of relative axial position. Only molecules that lasted at least 7 frames contribute to the distribution. *Bottom:* Distribution of axial projections on the same relative scale. B) Same as panel A, but for ribosomes imaged after Cam treatment. C) Same as panel A, but for EF-P–mEos2 molecules imaged in cells of length 3.5–4.5 μm after 3 hr of rifampicin treatment. D) Same as panel C, but for ribosomes imaged after Rif treatment.

**Figure S2.** Localization probability density maps of EF-P–mEos2 expressed from plasmid in normal growth conditions. A composite of images taken at 2 ms/frame from cells with lengths between 3.5–4.5 µm. Only molecules that lasted at least 7 frames contribute to the axial distribution. The axial distribution shows a three peak distribution similar to that of ribosomes under same imaging conditions.

**Figure S3.** A) *Red:* Experimental probability distribution of single-step displacements taken by EF-P–mEos2 molecules in 2 ms after 30 min of chloramphenicol treatment. *Black:* The best fit to a static two-state model (without transitions) with *D_slow_* constrained. Model parameters: *D_slow_* = 0.2 µm^2^/s (σ_slow_ = 50 nm), *f_slow_* = 0.45, *D_fast_* = 1.2 µm^2^/s (σ_fast_ = 90 nm), *f_fast_* = 0.55, with χ_ν_^2^ = 1.2. The slow and fast components are shown as dashed lines as labeled. B) *Red:* Experimental probability distribution of single-step displacements taken by EF-P–mEos2 molecules in 2 msafter 3 hr of rifampicin treatment. *Black:* The best unconstrained two-state model fit. Model parameters: *D_slow_* = 4.6 µm^2^/s (σ_slow_ = 75 nm), *f_slow_* = 0.55, *D_fast_* = 8 µm^2^/s (σ_fast_ = 150 nm), *f_fast_* = 0.45, with χ_ν_^2^ = 1.5. The individual slow and fast components are shown in dashed lines of blue and green respectively.

**Figure S4.** A)Mean square displacement plot, MSD(τ), for EF-P–mEos2 (red circles) and for ribosomes (30S–mEos2 labeling, black circles). Trajectories are truncated to six steps; error estimates are ± 1 σ of the MSD values. Lines are drawn through first two data, yielding diffusion coefficient estimates of 3.5 µm^2^/s and 0.5 µm^2^/s, respectively. B) Mean square displacement plot, MSD(τ), for EF-P–mEos2 after 3 hr of treatment with Rif (red circles) and the mutant form EF-P^K34A^–mEos2 in normal growth conditions (black circles). Trajectories are truncated to six steps; error estimates are ± 1 σ of the MSD values. Lines are drawn through first two data, yielding diffusion coefficient estimates of 6.7 µm^2^/s and 4.6 µm^2^/s, respectively.

**Figure S5.** A) For EF-P–mEos2 in normal growth conditions, probability distribution of the mean of six successive one-step estimates of the diffusion coefficient, *P*(*<D>_6-step_*). Cut-off values of *D* = 1.56 µm^2^/s and *D* = 7.8 µm^2^/s select the 10% slowest and the 10% fastest mean values. B) Mean square displacement plots, MSD(τ), for the fastest 10% of EF-P–mEos2 trajectories (red circles) and the slowest 10% (black circles). Error estimates are ± 1 σ of the MSD values. The slope of the first two data points yields diffusion coefficient estimates of 8.65 µm^2^/s and 0.66 μm^2^/s, respectively. Intercepts are used to estimate dynamic localization errors σ_fast_and σ_slow_.

**Figure S6.** Experimental probability distribution of the mean of six successive single-step displacements of EF-P–mEos2 trajectories truncated to 6 steps, *P*(*<r>*_6_) (bold line). Dashed lines are simulated results for two-state models with binding-unbinding kinetics. All simulations use the model parameters *D_slow_* = 0.2 µm^2^/s (σ_slow_ = 50 nm), *f_slow_* = 0.3, *D_fast_* = 4.3 µm^2^/s (σ_fast_ = 75 nm), *f_fast_* = 0.7, taken from the best fit to the single-step *P_EF-P_*(*r*) as in Fig. 4A. The ratio τ_free_ / τ_bound_ = 7/3 is fixed by the best-fit fractions. The best fit has τ_free_ = 16 ms (and τ_bound_ = 7 ms), with __^2^ = 1.4, as shown in Fig. 4. The simulation results shown for τ_free_ = 11 ms (χ_ν_^2^ = 1.95) and 20 ms (χ_ν_^2^ = 2.12) set the error bars on τ_free_. Also shown are simulations for τ_free_ = 0.1 ms (fast exchange limit compared with 2-ms camera frame time) and 100 ms (slow exchange limit), which give worse fits.

**Figure S7.** Reduced chi-square values __^2^ for fits to *P_EF-P_*(*r*) using different parameter sets.Three slices through the 3D grid of χ_ν_^2^ values (with fitting parameters *D_slow_*, *D_fast_*, and *f_slow_*) obtained in modeling the experimental distribution of one-step displacements in Fig. 4A. A) Slice through the plane with *D_slow_* = 0.2 μm/s; *D_fast_* and *f_slow_* vary. B) Slice through the plane with
*D_fast_*  = 4.3 μm^2^/s; *D_slow_* and *f_slow_*vary. C) Slice through the plane with *f_slow_* = 0.30; *D_slow_* and *D_fast_* vary. Regions outlined in blue have χ_ν_^2^ ≤ 1.5 and produce qualitatively poorer fits than those with χ_ν_^2^ ~ 1. Those regions were used for error estimates on best-fit parameters.


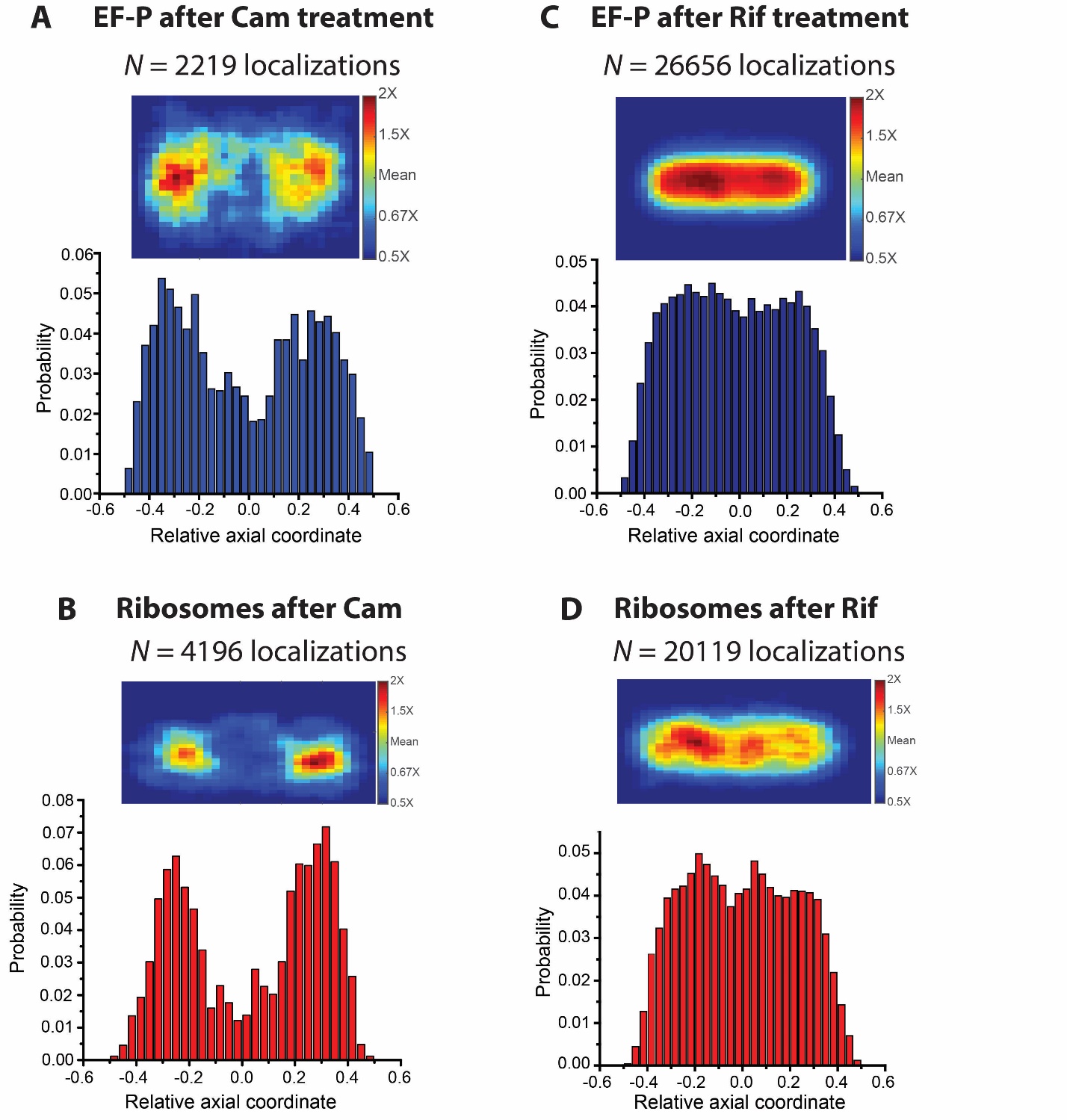


**Figure S1.**


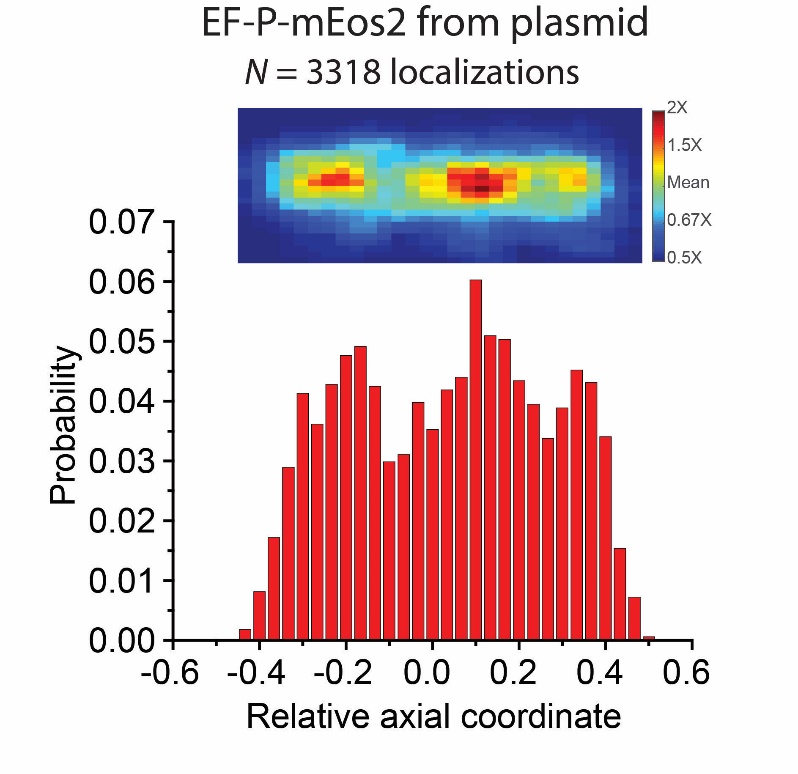


**Figure S2.**


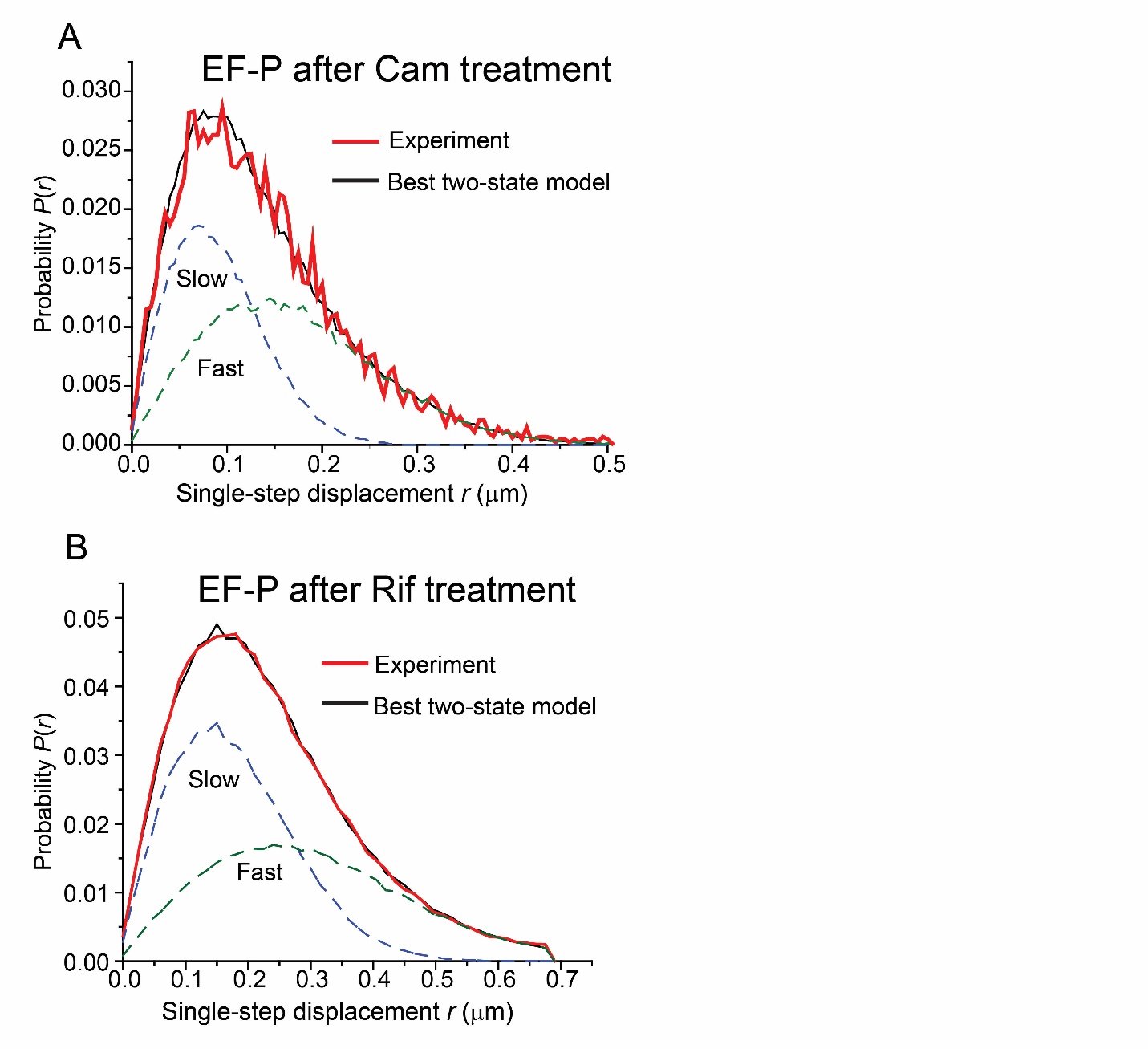


**Figure S3.**


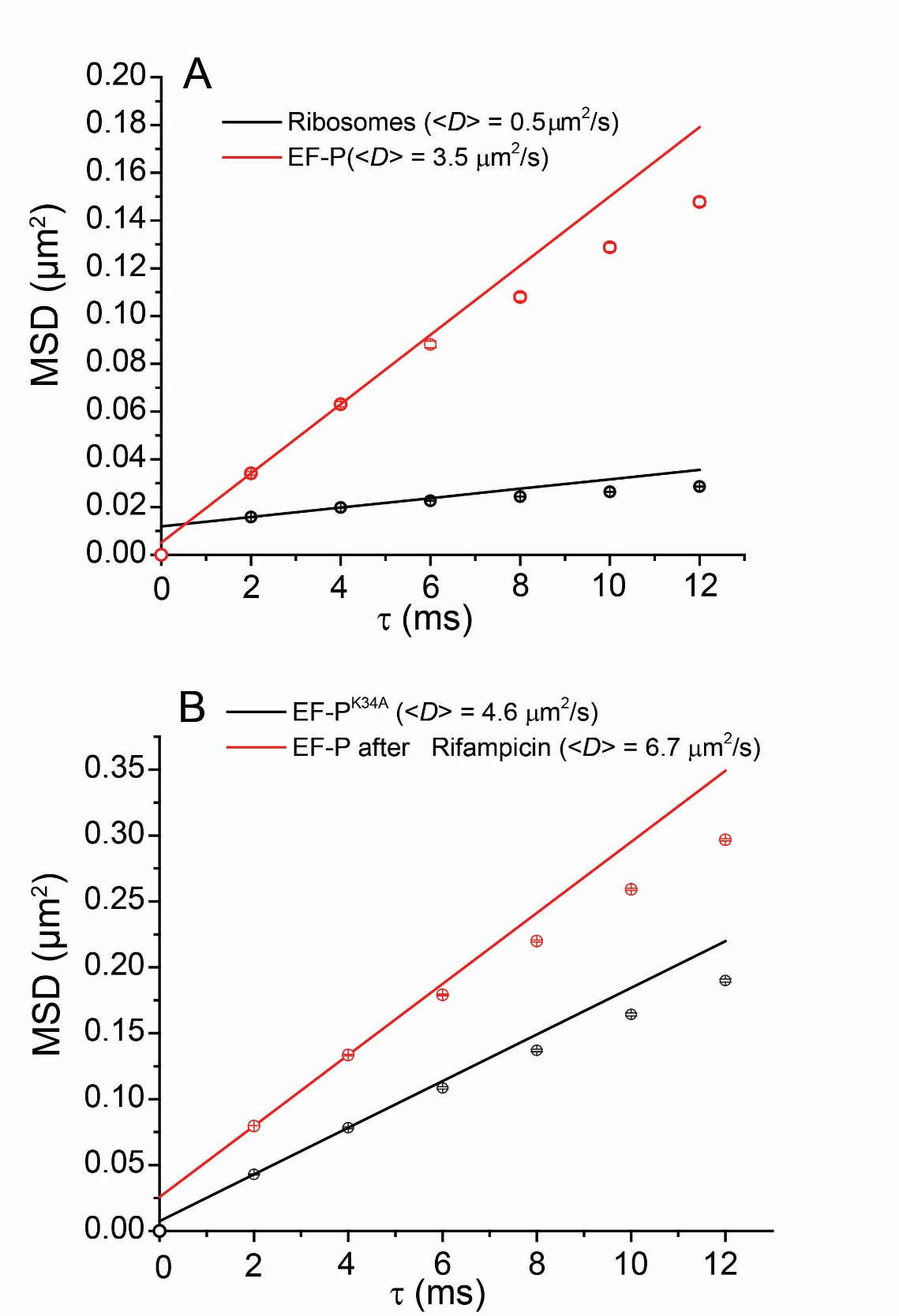


**Figure S4.**


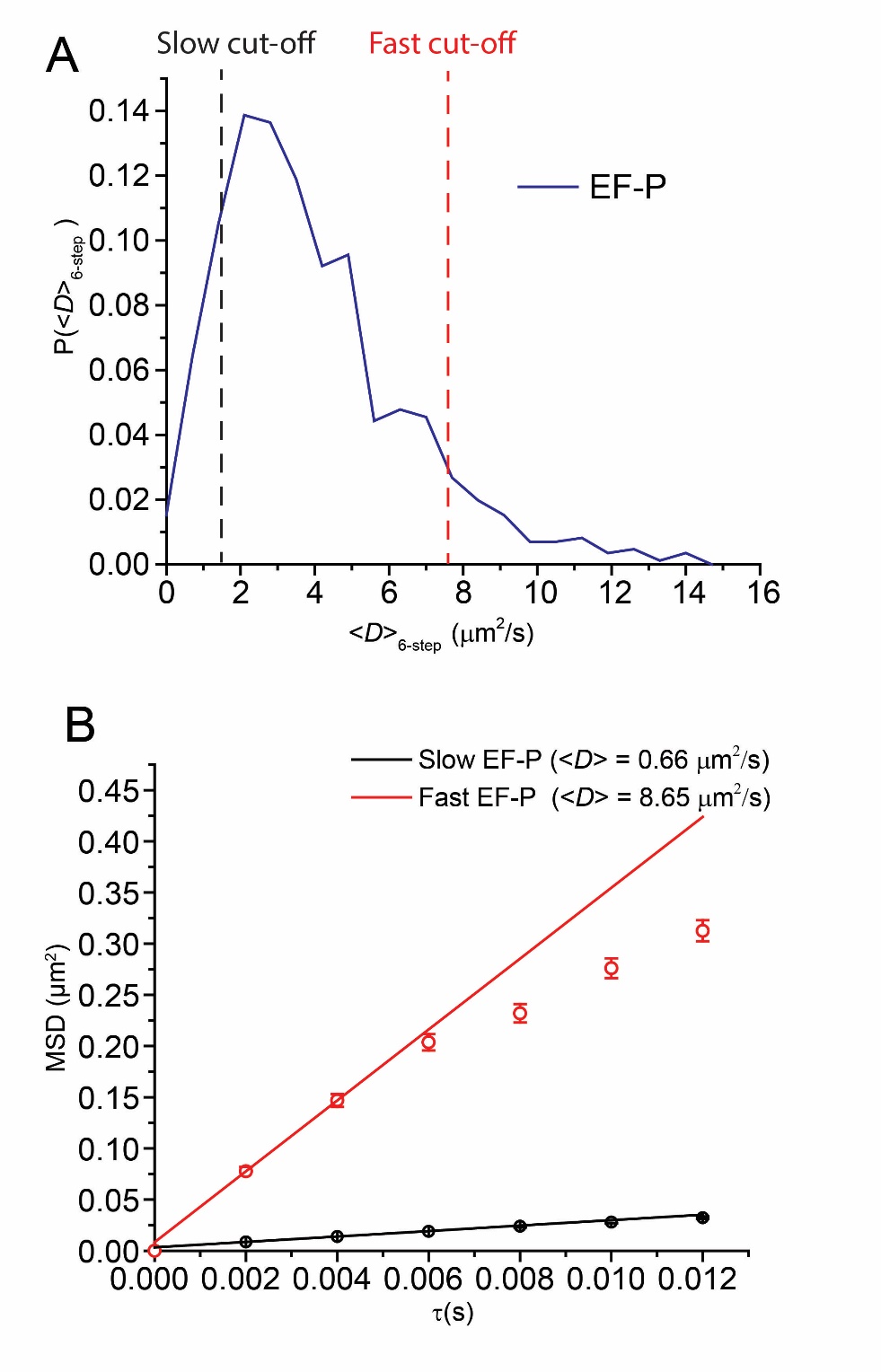


**Figure S5.**


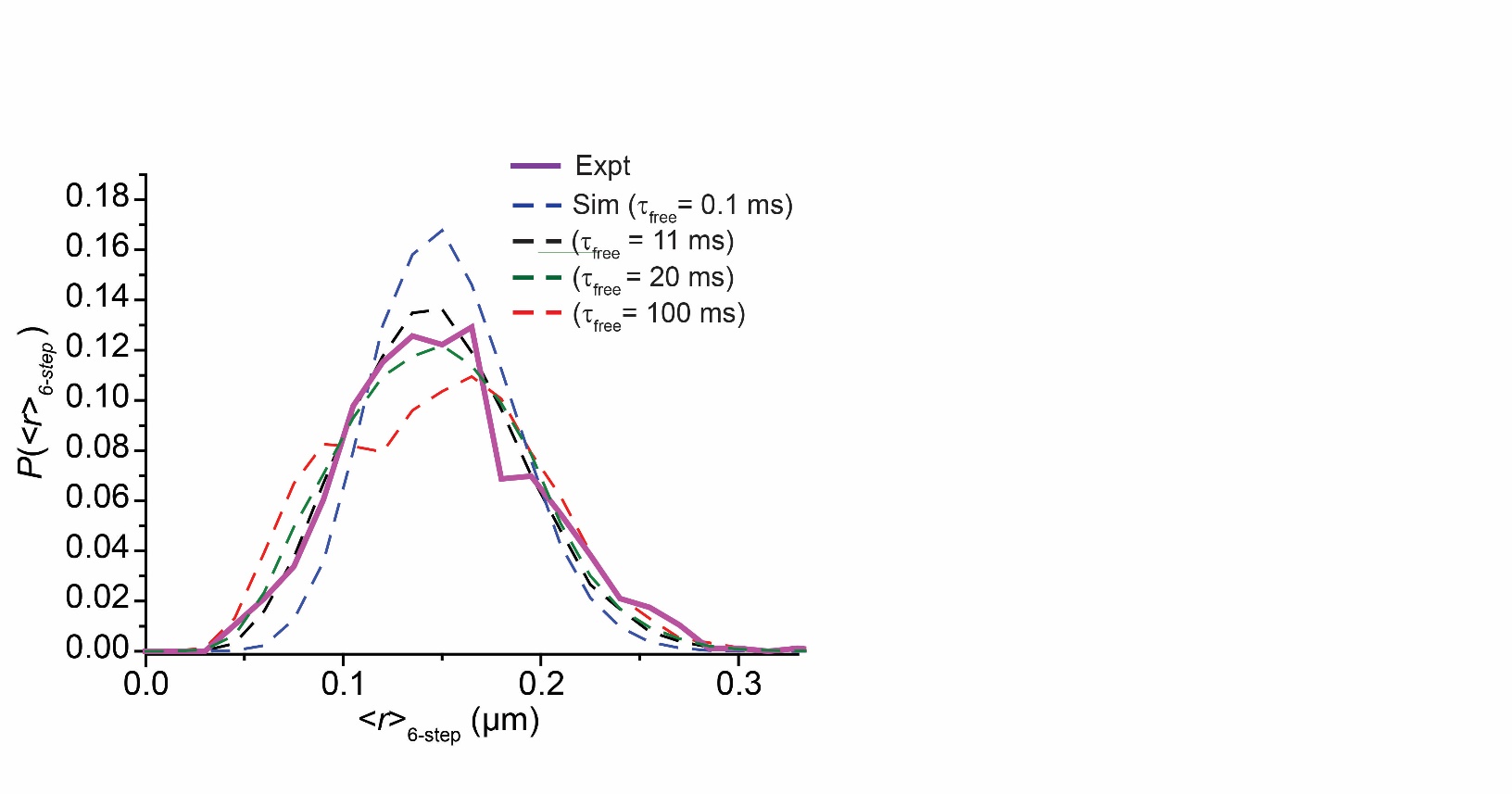


**Figure S6.**

**A. *D_slow_* = 0.2 μm^2^-s^-1^**


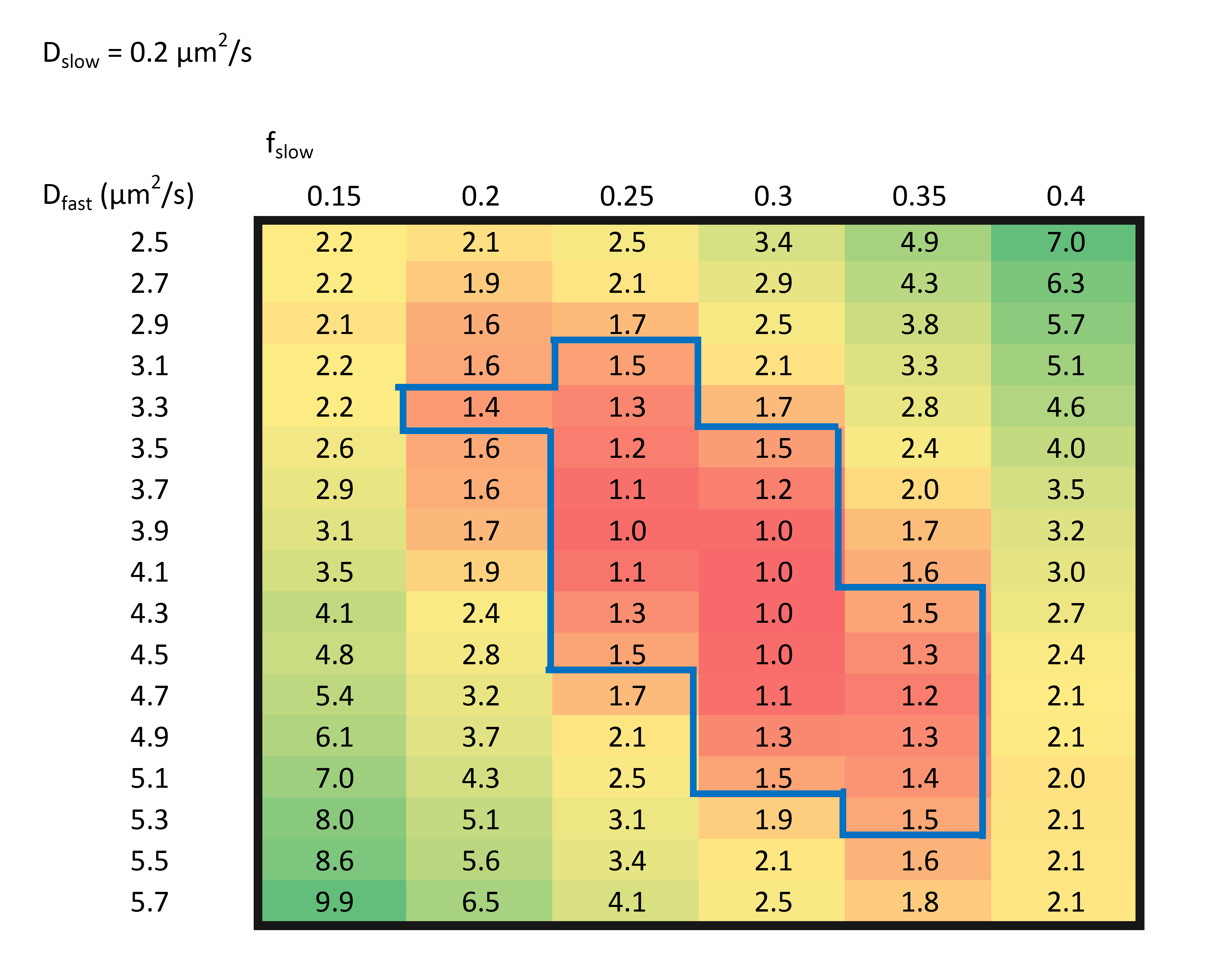


**B. *D_fast_* = 4.3 μm^2^-s^-1^**


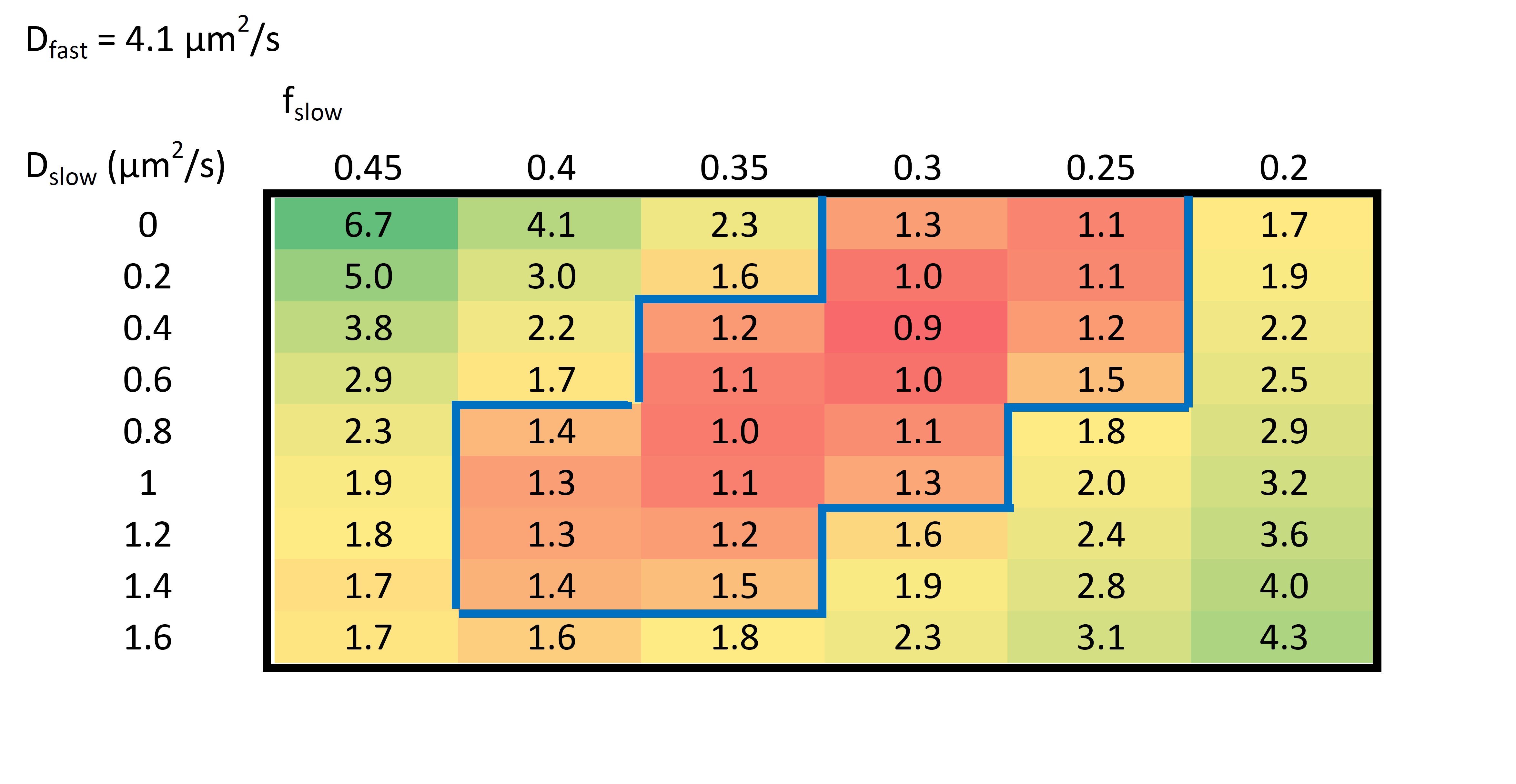


**C. *f_slow_* = 0.30**


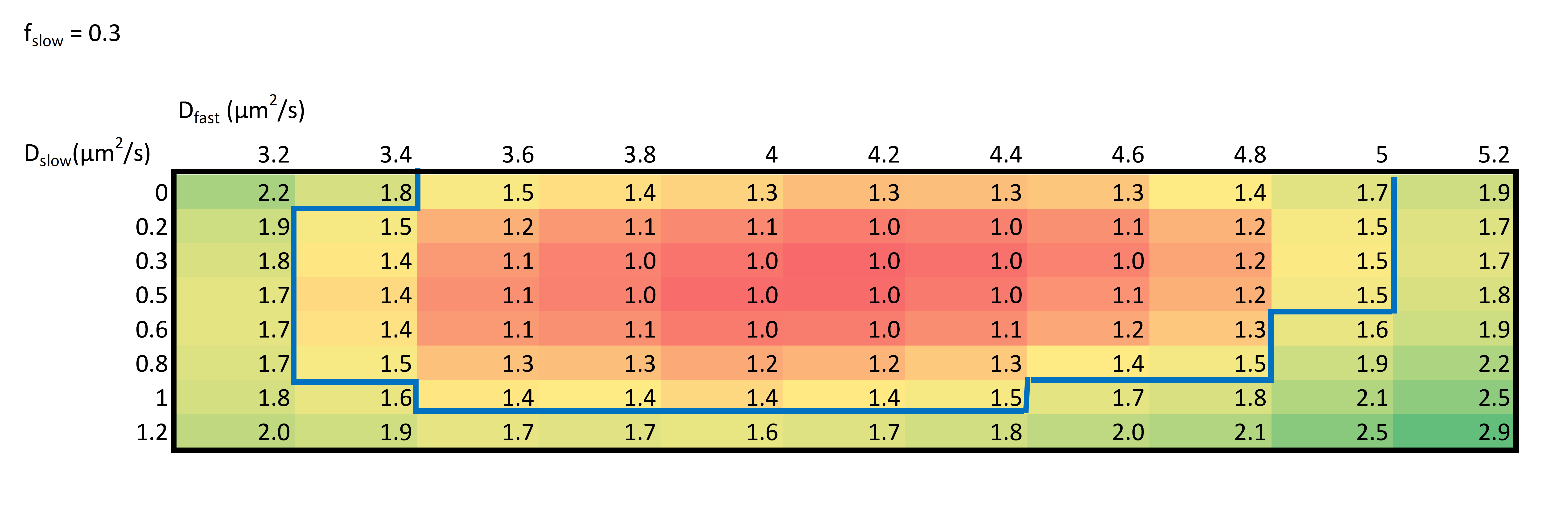


**Figure S7.**

**Supplemental References**

1. Bakshi S, Bratton BP, Weisshaar JC.2011. Subdiffraction-limit study of Kaede diffusion and spatial distribution in live Escherichia coli. Biophys J 101:2535-44.

2. Michalet X.2010. Mean Square Displacement Analysis of Single-Particle Trajectories with Localization Error: Brownian Motion in Isotropic Medium. Physical review E, Statistical, nonlinear, and soft matter physics 82:041914-041914.

3. Thompson RE, Larson DR, Webb WW.2002. Precise nanometer localization analysis for individual fluorescent probes. Biophys J 82:2775-2783.

4. Bakshi S, Dalrymple RM, Li W, Choi H, Weisshaar JC.2013. Partitioning of RNA polymerase activity in live *Escherichia coli* from analysis of single-molecule diffusive trajectories. Biophys J 105:2676-2686.

5. Bakshi S, Siryaporn A, Goulian M, Weisshaar JC.2012. Superresolution imaging of ribosomes and RNA polymerase in live Escherichia coli cells. Mol Microbiol 85:21-38.

6. Stracy M, Jaciuk M, Uphoff S, Kapanidis AN, Nowotny M, Sherratt DJ, Zawadzki P.2016. Single-molecule imaging of UvrA and UvrB recruitment to DNA lesions in living Escherichia coli. Nature Comm 7:12568.

7. Chen T-Y, Santiago AG, Jung W, Krzemiński Ł, Yang F, Martell DJ, Helmann JD, Chen P.2015. Concentration- and chromosome-organization-dependent regulator unbinding from DNA for transcription regulation in living cells. Nature Comm 6:7445.

8. Li W, Bouveret E, Zhang Y, Liu K, Wang JD, Weisshaar JC.2016. Effects of amino acid starvation on RelA diffusive behavior in live Escherichia coli. Mol Microbiol 99:571-85.

9. Das R, Cairo CW, Coombs D.2009. A hidden Markov model for single particle tracks quantifies dynamic interactions between LFA-1 and the actin cytoskeleton. PLoS Comput Biol 5:e1000556.
