# Supplementary figures and images for: Spatial Distribution and Ribosome-Binding Dynamics of EF-P in Live Escherichia coli
Source: mBio. 2017 Jun 6;8(3):e00300-17. doi: 10.1128/mBio.00300-17 (PMC5461404; doi:10.1128/mBio.00300-17)

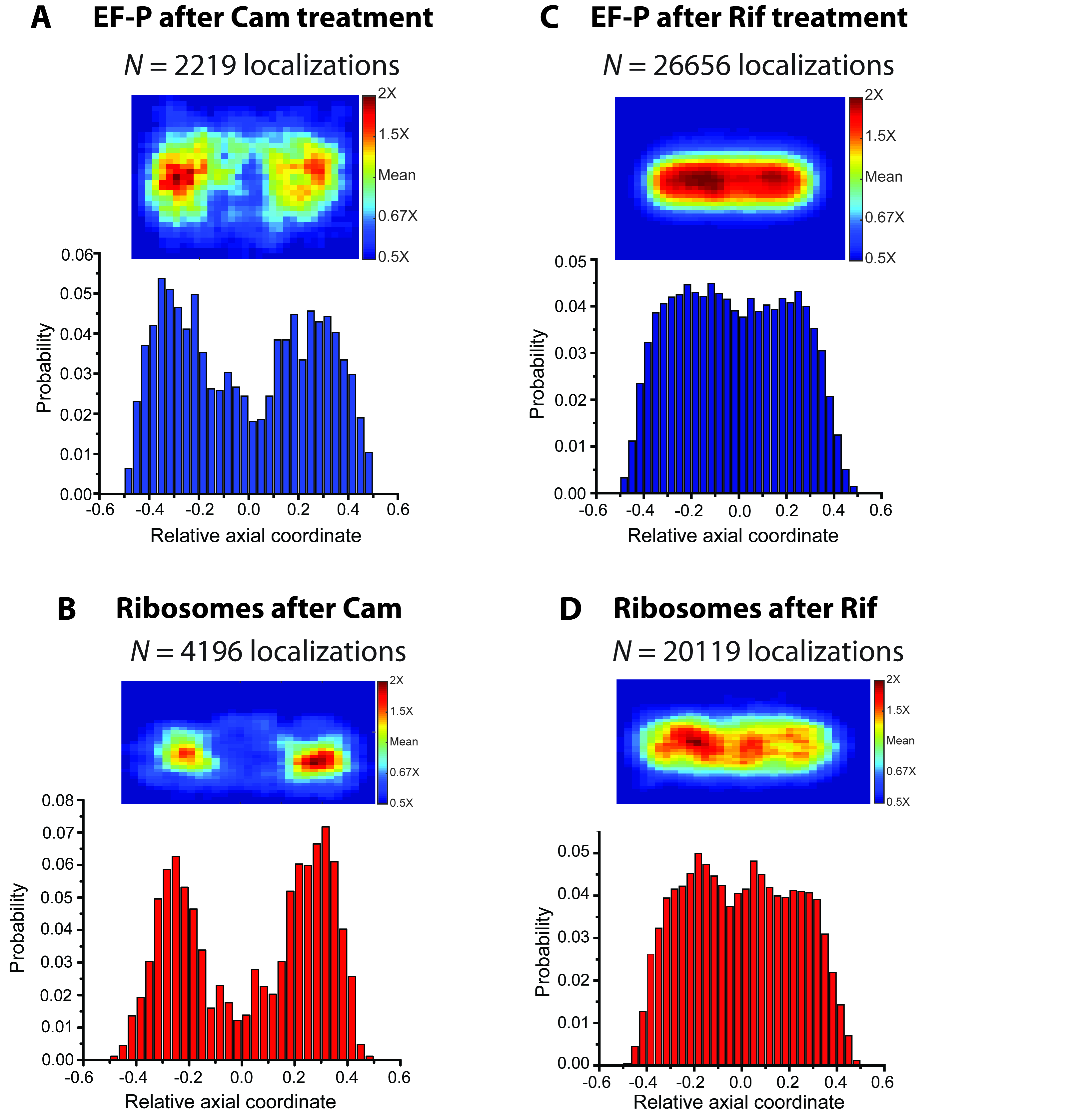

Supplement: FIG S1 [file mbo003173332sf1.tif]

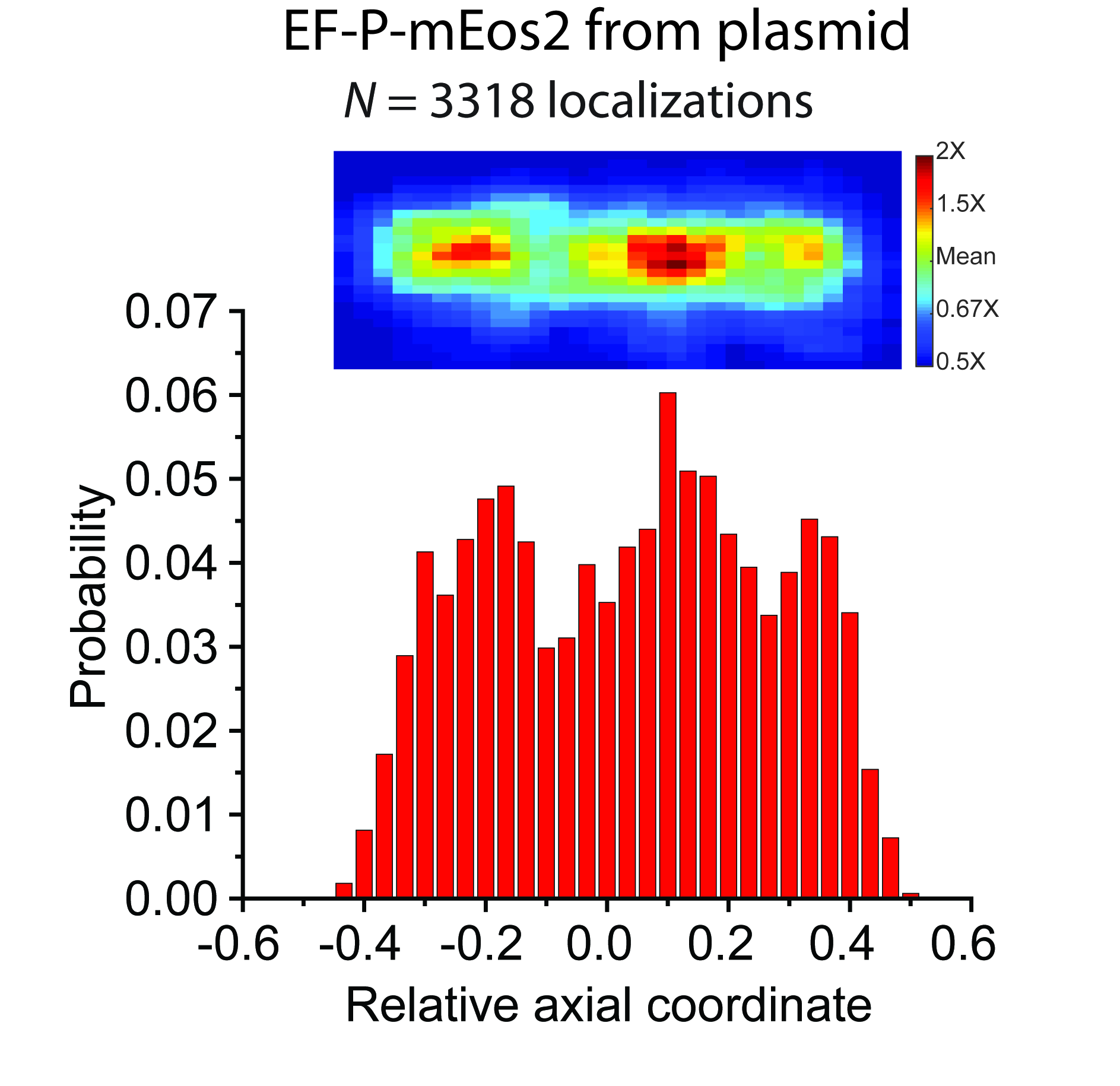

Supplement: FIG S2 [file mbo003173332sf2.tif]

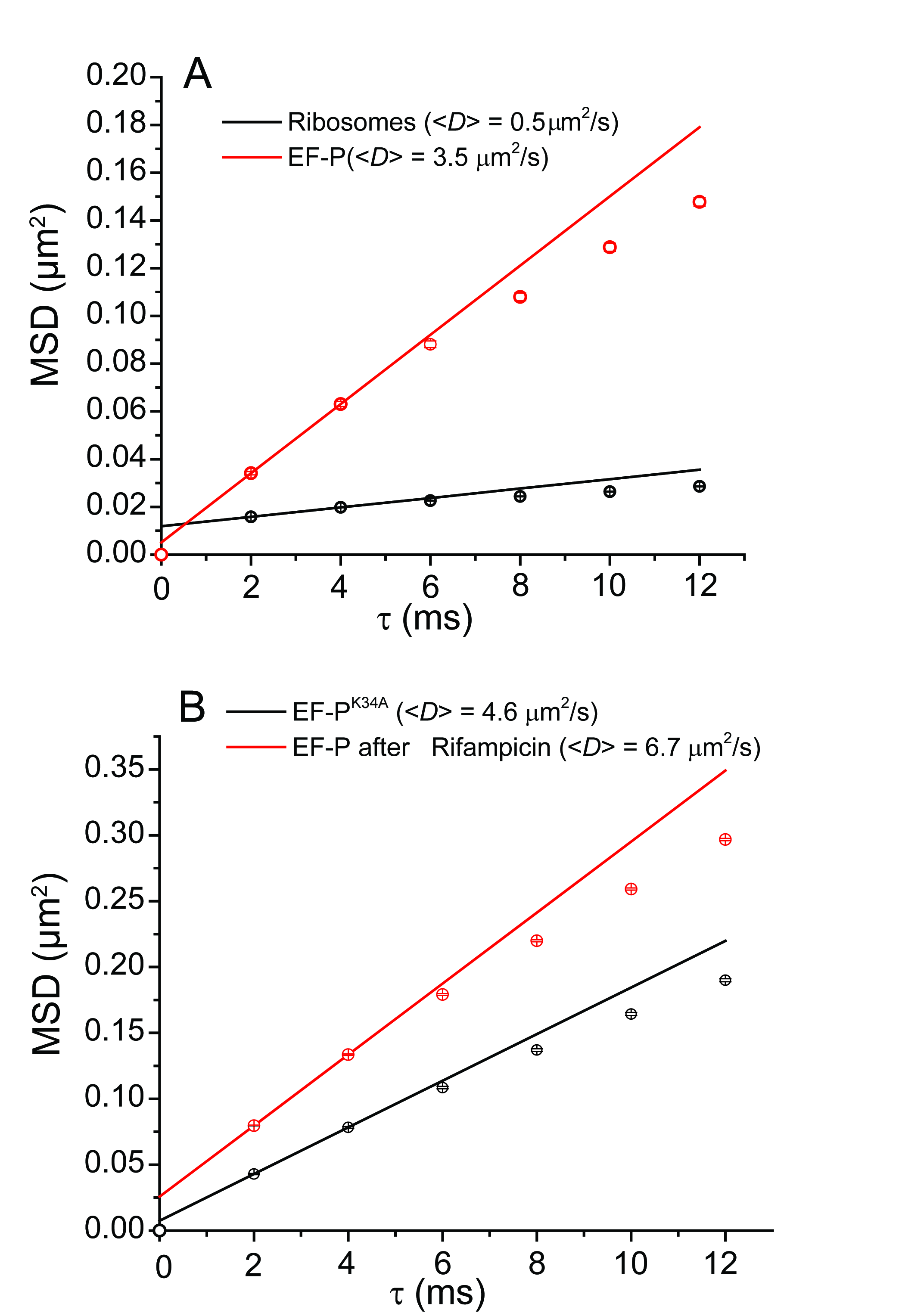

Supplement: FIG S4 [file mbo003173332sf4.tif]

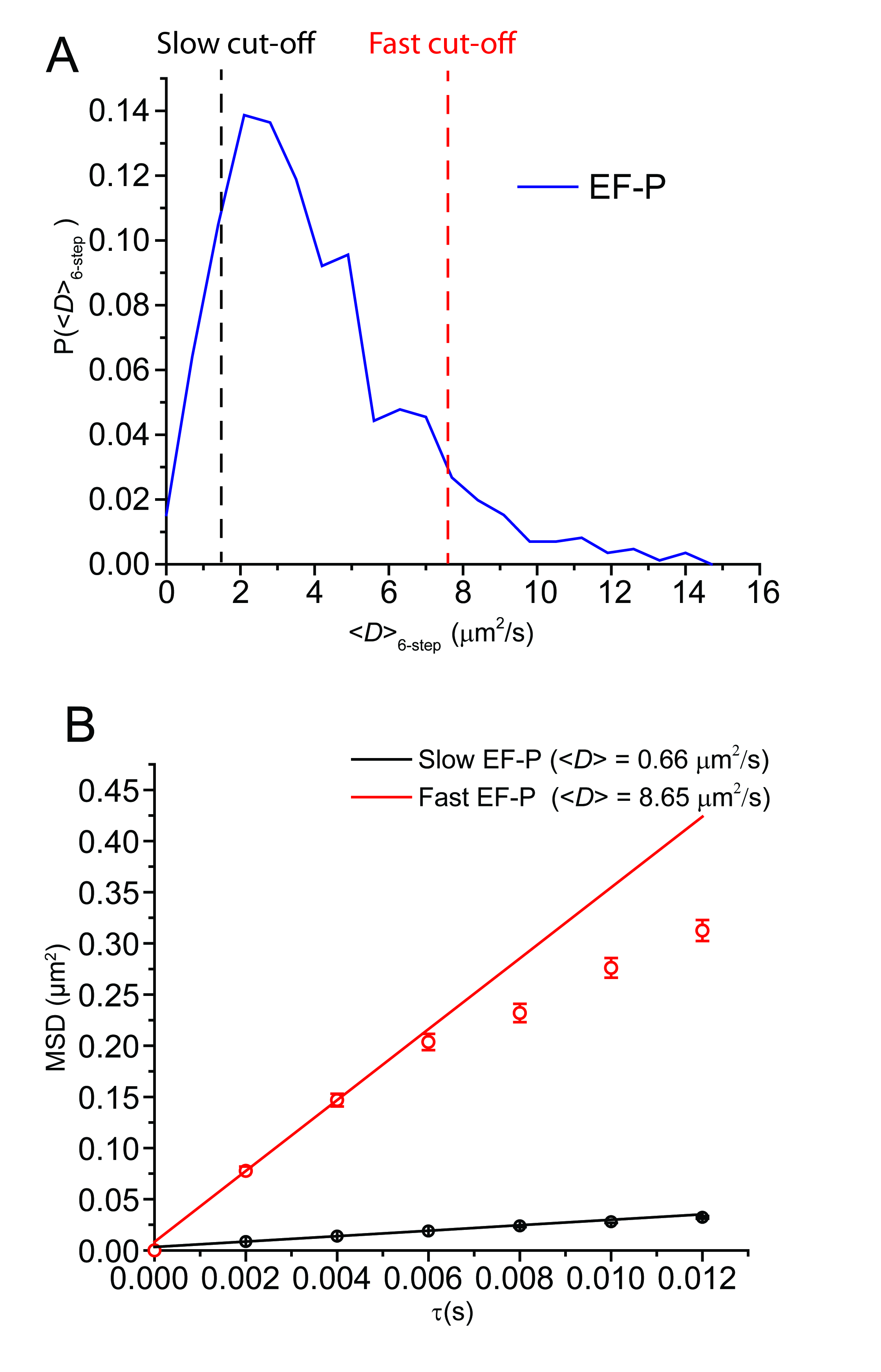

Supplement: FIG S5 [file mbo003173332sf5.tif]

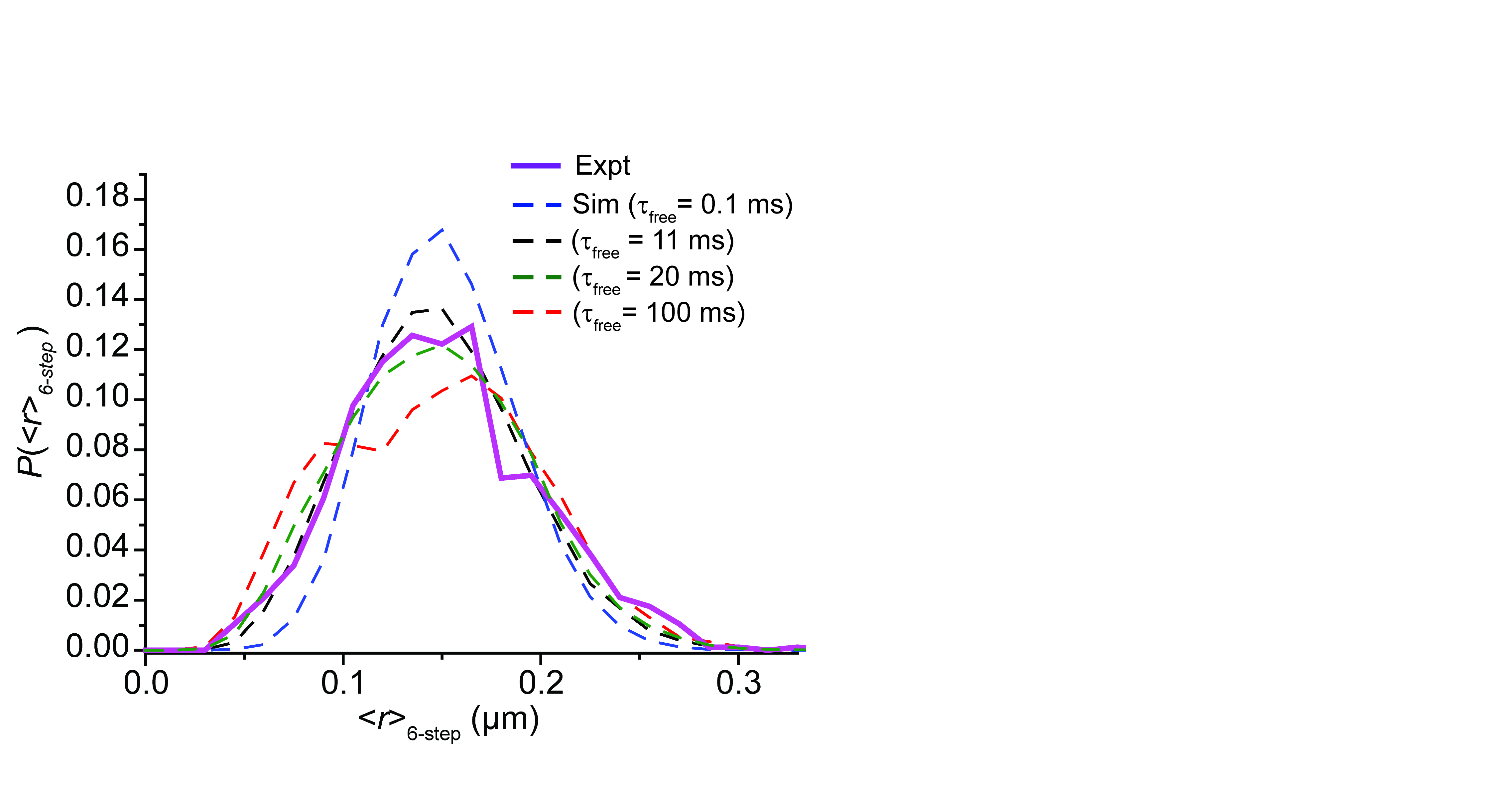

Supplement: FIG S6 [file mbo003173332sf6.tif]

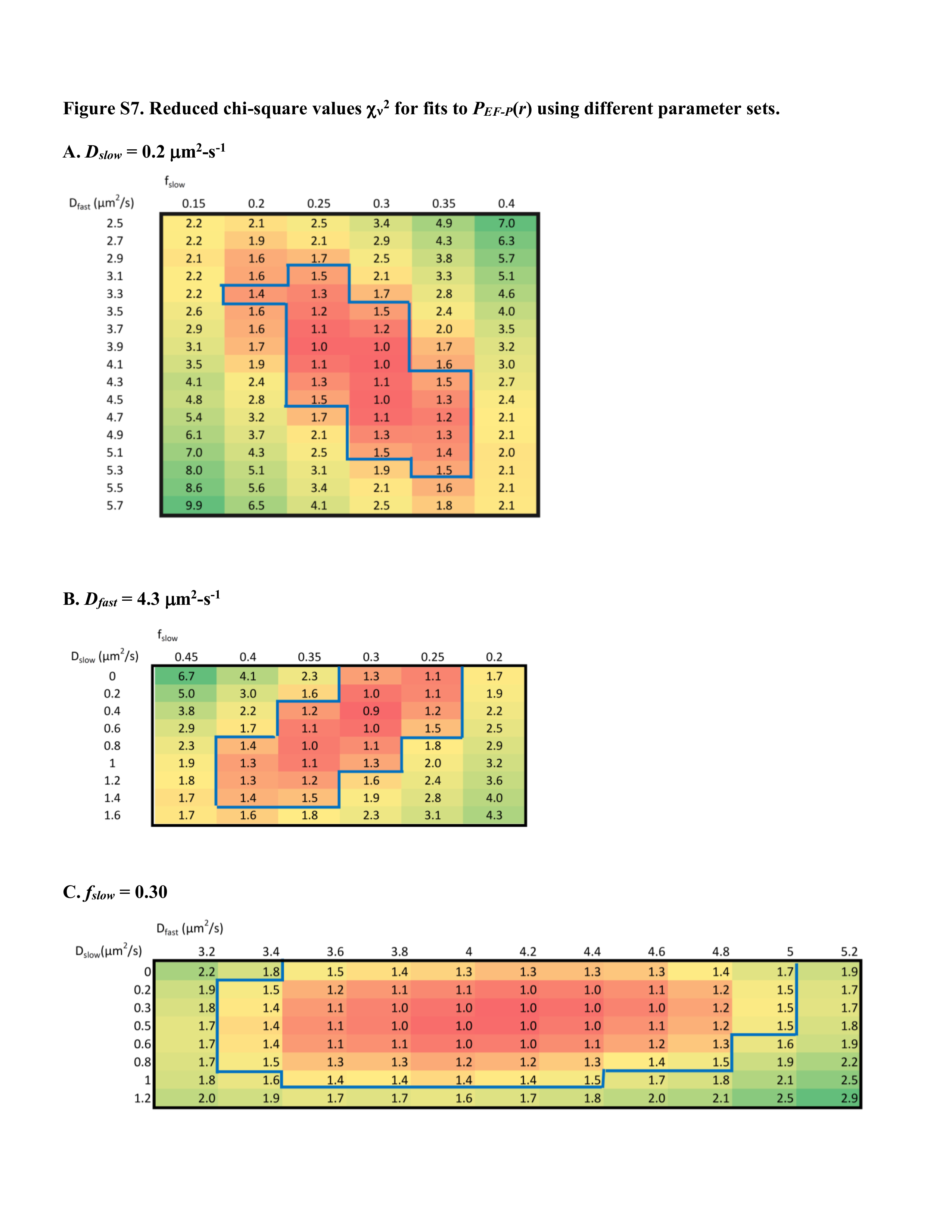

Supplement: FIG S7 [file mbo003173332sf7.tif]
