# Supplementary material for: Spatial Distribution and Ribosome-Binding Dynamics of EF-P in Live Escherichia coli
Source: mBio. 2017 Jun 6;8(3):e00300-17. doi: 10.1128/mBio.00300-17 (PMC5461404; doi:10.1128/mBio.00300-17)
Supplement: TABLE S2 [file mbo003173332st2.docx]

**Table S2. Summary of range of fitting searches for *P*(*r*) for different species and imaging conditions.**

| **Imaging conditions** | **Range of *D_slow_*  (μm^2^/s)** | **Range of *D_fast_* (μm^2^/s)** | **Best *D_slow_***  **(μm^2^/s)** | **Best *D_fast_***  **(μm^2^/s)** | **Best f_slow_** |
| --- | --- | --- | --- | --- | --- |
| **Ribosome** | 0-0.9  (σ_slow_ = 40 nm) | 0-1  (σ_fast_ = 75 nm) | 0.2 | 0.8 | 0.65 |
| **EF-P** | Constrained to 0.2 (σ_slow_ = 50 nm) | 1-15  (σ_fast_ = 75 nm) | Constrained to 0.2 | 4.3 | 0.30 |
| **EF-P under Cam** | Constrained to 0.2 (σ_slow_ = 50 nm) | 1-15  (σ_fast_ = 90 nm) | Constrained to 0.2 | 1.2 | 0.45 |
| **EF-P under Rif** | 1-10 (σ_slow_ = 125 nm) | 1-10 (σ_fast_ = 150 nm) | 4.6 | 8 | 0.55 |
| **EF-P^K34A^** | 1-10 (σ_slow_ = 50 nm) | 1-15 (σ_fast_ = 90 nm) | 3.2 | 9.7 | 0.65 |
